# The V2 domain of HIV gp120 mimics an interaction between CD4 and integrin ⍺_4_β_7_

**DOI:** 10.1371/journal.ppat.1011860

**Published:** 2023-12-08

**Authors:** Donald Van Ryk, Sinmanus Vimonpatranon, Joe Hiatt, Sundar Ganesan, Nathalie Chen, Jordan McMurry, Saadiq Garba, Susie Min, Livia R. Goes, Alexandre Girard, Jason Yolitz, Isabella Licavoli, Danlan Wei, Dawei Huang, Marcelo A. Soares, Elena Martinelli, Claudia Cicala, James Arthos

**Affiliations:** 1 Laboratory of Immunoregulation, National Institute of Allergy and Infectious Diseases, Bethesda, Maryland, United States of America; 2 Department of Retrovirology, Armed Forces Research Institute of Medical Sciences–United States Component, Bangkok, Thailand; 3 Biological Imaging Section, National Institute of Allergy and Infectious Diseases, Bethesda, Maryland, United States of America; 4 Oncovirology Program, Instituto Nacional de Câncer, Rio de Janeiro, Rio de Janeiro, Brazil; 5 Lymphoid Malignancies Branch, National Cancer Institute, Bethesda, Maryland, United States of America; 6 Department of Genetics, Universidade Federal do Rio de Janeiro, Rio de Janeiro, Brazil; 7 Department of Cell and Developmental Biology, Feinberg School of Medicine, Northwestern University, Chicago, Illinois, United States of America; Loyola University Chicago, UNITED STATES

## Abstract

The CD4 receptor, by stabilizing TCR-MHC II interactions, plays a central role in adaptive immunity. It also serves as the HIV docking receptor. The HIV gp120 envelope protein binds directly to CD4. This interaction is a prerequisite for viral entry. gp120 also binds to ⍺_4_β_7_, an integrin that is expressed on a subset of memory CD4^+^ T cells. HIV tropisms for CD4^+^ T cells and gut tissues are central features of HIV pathogenesis. We report that CD4 binds directly to ⍺_4_β_7_ in a dynamic way, consistent with a cis regulatory interaction. The molecular details of this interaction are related to the way in which gp120 interacts with both receptors. Like MAdCAM-1 and VCAM-1, two recognized ligands of ⍺_4_β_7_, the binding interface on CD4 includes 2 sites (1° and accessory), distributed across its two N-terminal IgSF domains (D1 and D2). The 1° site includes a sequence in the G β-strand of CD4 D2, KIDIV, that binds directly to ⍺_4_β_7_. This pentapeptide sequence occurs infrequently in eukaryotic proteins. However, a closely related and conserved sequence, KLDIV, appears in the V2 domain of gp120. KLDIV mediates gp120-⍺_4_β_7_ binding. The accessory ⍺_4_β_7_ binding site on CD4 includes Phe^43^. The Phe^43^ aromatic ring protrudes outward from one edge of a loop connecting the C’C” strands of CD4 D1. Phe^43^ is a principal contact for HIV gp120. It interacts with conserved residues in the recessed CD4 binding pocket. Substitution of Phe^43^ abrogates CD4 binding to both gp120 and ⍺_4_β_7_. As such, the interactions of gp120 with both CD4 and ⍺_4_β_7_ reflect elements of their interactions with each other. These findings indicate that gp120 specificities for CD4 and ⍺_4_β_7_ are interrelated and suggest that selective pressures which produced a CD4 tropic virus that replicates in gut tissues are linked to a dynamic interaction between these two receptors.

## Author summary

Integrins modulate cell trafficking and provide costimulatory signals to leukocytes. An increasing number have also been shown to interact in cis with immune receptors. Formation of these multi-receptor complexes is dynamic and typically serves a regulatory function. We report a cis interaction between ⍺_4_β_7_, the gut-homing integrin and CD4, a receptor that plays a central role in the adaptive immune response. CD4-⍺_4_β_7_ interactions share features with MAdCAM-1 and VCAM-1, two natural ligands that engage ⍺_4_β_7_ in trans. All three receptors engage ⍺_4_β_7_ through two discreet sites distributed across their two N-terminal IgSF domains. Specific features of the two ⍺_4_β_7_ binding sites on CD4 are related to HIV pathogenesis in a unique and informative way. HIV gp120 binds to both CD4 and ⍺_4_β_7_. A key finding of this report is that specific elements of gp120-CD4 and gp120-⍺_4_β_7_ interactions reflect the interaction of these two receptors with each other. We conclude that the selective pressures that resulted in a virus which infects and depletes gut CD4^+^ T cells are linked to CD4-⍺_4_β_7_ cis interactions.

## Introduction

The discovery that the HIV gains entry into CD4^+^ T cells by binding directly to the CD4 receptor [1–3], followed by the determination that neutralizing antibodies are directed exclusively to the gp120/41 envelope protein [4], provided a rationale for developing an HIV vaccine immunogen that would elicit CD4 binding site (CD4bs) antibodies. Features intrinsic to envelope-CD4 interactions suppress the elicitation of such antibodies [5,6]. CD4 is comprised of 4 tandemly arrayed Ig -like ectodomains (D1-D4) followed by short transmembrane and cytoplasmic domains [7]. As a first step, gp120 engages a phenylalanine at position 43 (Phe^43^) in D1 [8,9]. The Phe^43^ aromatic sidechain protrudes from the edge of an upward facing loop near the apex of CD4 [10]. The CD4bs on gp120 that accommodates Phe^43^ appears as a spherical cavity. Insertion of Phe^43^ into this cavity precipitates a significant conformational reorganization of the gp120/41 prefusion trimer [11–14]. This includes the decoupling of the V2 and V3 domains, which in turn facilitates V3-coreceptor engagement and, ultimately, virus fusion with the cell membrane [14–16]. Although we understand gp120-CD4 interactions in detail, the selective pressures that produced this interaction in the first instance remain unclear.

In considering the selective pressures that resulted in a CD4bs on gp120 that engages Phe^43^ it is useful to consider CD4’s normal role. Together with Lck, CD4 transiently migrates into the TCR where it binds to MHC II on APCs and stabilizes TCR interactions [17–19]. Of note, gp120 and MHC II engage CD4 in distinct ways [20]. Substitution of Phe^43^ eliminates gp120 reactivity but does not disrupt CD4-MHC II interactions [21,22]. Given its exposed presentation, it would appear that Phe^43^ serves some function. Identifying a natural role for this aa might begin to reveal the selective pressures which resulted in the specialized interaction between gp120 and CD4.

In addition to CD4, the HIV envelope engages several ancillary receptors that may facilitate mucosal transmission. These include DC-SIGN [23], Siglec-1 [24], and the mannose receptor [25]. Because it is difficult to model mucosal transmission in vitro, and even in non-human primates (NHP), the importance of these interactions is subject to debate. Integrin ⍺_4_β_7_ (⍺_4_β_7_) is an additional ancillary receptor that we and others have shown binds gp120 [26–29]. ⍺_4_β_7_ mediates T cell trafficking to gut associated lymphoid tissues (GALT) via interactions with two adhesion receptors, MAdCAM-1 and VCAM-1, that are expressed on high endothelial venules that line gut tissues [30,31]. ⍺_4_β_7_ is not required for HIV entry or infection, and not all recombinant gp120s bind to it. We have speculated that ⍺_4_β_7_ provides a way for HIV to increase transmission efficiency by enhancing access to the plentiful supply of activated CD4^+^ CCR5^+^ T cells in GALT. In support of this proposition, higher frequencies of ⍺_4_β_7_^high^ CD4^+^ memory T cells correlate with increased susceptibility to HIV acquisition [32]. Additionally, ⍺_4_β_7_^high^ CD4^+^ memory T cells collected from HIV infected subjects in the early stages of infection (FIEBIG II/III) are enriched in HIV proviral DNA, relative to other CD4^+^ T cell subsets [33], and this same subset is selectively infected and depleted from SIV infected macaques [34]. Finally, an ⍺_4_β_7_ -specific mAb was shown to block vaginal transmission of SIV in a repeated low-dose challenge model [35].

In a previous study we determined that ⍺_4_β_7_ clusters with CD4 and CCR5 on the surfaces of some ⍺_4_β_7_^high^ CD4^+^ effector memory T cells [36]. This is puzzling. ⍺_4_β_7_ does not play a role MHC II-CD4/TCR interactions. Conversely, there is no direct role for CD4 in ⍺_4_β_7_ -mediated T cell trafficking. In this report we show that ⍺_4_β_7_ binds directly to CD4 in cis. In this way it adds to the list of integrins that engage counter receptors in both trans (opposing cell) and cis (same cell) [37] (S1A Fig). We find that elements of this interaction are incorporated into the HIV envelope as a means of recognizing both receptors. These findings address the selective pressures that underlie the specific affinity of gp120 for CD4.

## Results

### HIV gp120 enhances clustering of CD4 and ⍺_4_β_7_

CD4, CCR5 and ⍺_4_β_7_ colocalize on the surfaces of primary CD4^+^ T cells derived from both colon biopsies and blood derived CD4^+^ T cells cultured in retinoic acid (RA) [36]. To determine whether CD4 and ⍺_4_β_7_ come into close-proximity, we carried out a flow-cytometry based proximity ligation assays (PLA) on freshly isolated CD4^+^ T cells using CD4 and β_7_ mAbs as probes (S1B Fig). Positive PLA signals indicate a proximity <40nm. Cells were costained with a CD45RA mAb and vedolizumab to distinguish ⍺_4_β_7_^high^ memory from ⍺_4_β_7_^int^ naïve cell subsets. We observed positive PLA between CD4 and ⍺_4_β_7_ on both subsets, however the frequency of measurable PLA on ⍺_4_β_7_^high^ memory cells was ~2.5 greater than on naïve CD4^+^ T cells, indicating HIV gp120, like MAdCAM-1, can deliver costimulatory signals through ⍺_4_β_7_ in a way that promotes cellular proliferation and HIV infection [38,39]. This finding prompted us to ask whether gp120 -mediated signaling might influence CD4-⍺_4_β_7_ clustering. To this end, we employed fluorescence resonance energy transfer (FRET) measured by fluorescence lifetime imaging microscopy (FLIM). Primary CD4^+^ T cells were cultured in the presence of anti CD3, IL-2 and RA for 8 days and then placed on glass coverslips coated with HIV gp120 (stimulated) or without gp120 (unstimulated). Fluorescence lifetime measurements were performed on the CD4 donor mAb tagged with Alexa-488, in the presence or absence of an integrin β_7_ acceptor mAb (tagged with Alexa-546). FLIM values were acquired at 20’ and 60’ time points. Cells treated with gp120 showed a substantial reduction in lifetime signals at 20’ and a further reduction at 60’ (S1C and S1D Fig). These reductions in the lifetime of the donor indicate proximity of the CD4 and β_7_ probes, with an approximate FRETing distances of <15nm, indicating a direct interaction. In considering the role such an interaction might play in CD4^+^ T cell function, we note numerous reports of integrins regulating the activity of other cell surface receptors through direct cis interactions [37] (S1 Fig). A number of these cis interactions involve receptors that, like CD4, present tandem N-terminal Ig -like domains [40–44]. With these reports in mind, we sought to gather additional evidence for a direct physical interaction between CD4 and ⍺_4_β_7_.

### Specific binding of CD4 to ⍺_4_β_7_

**Fig 1:**
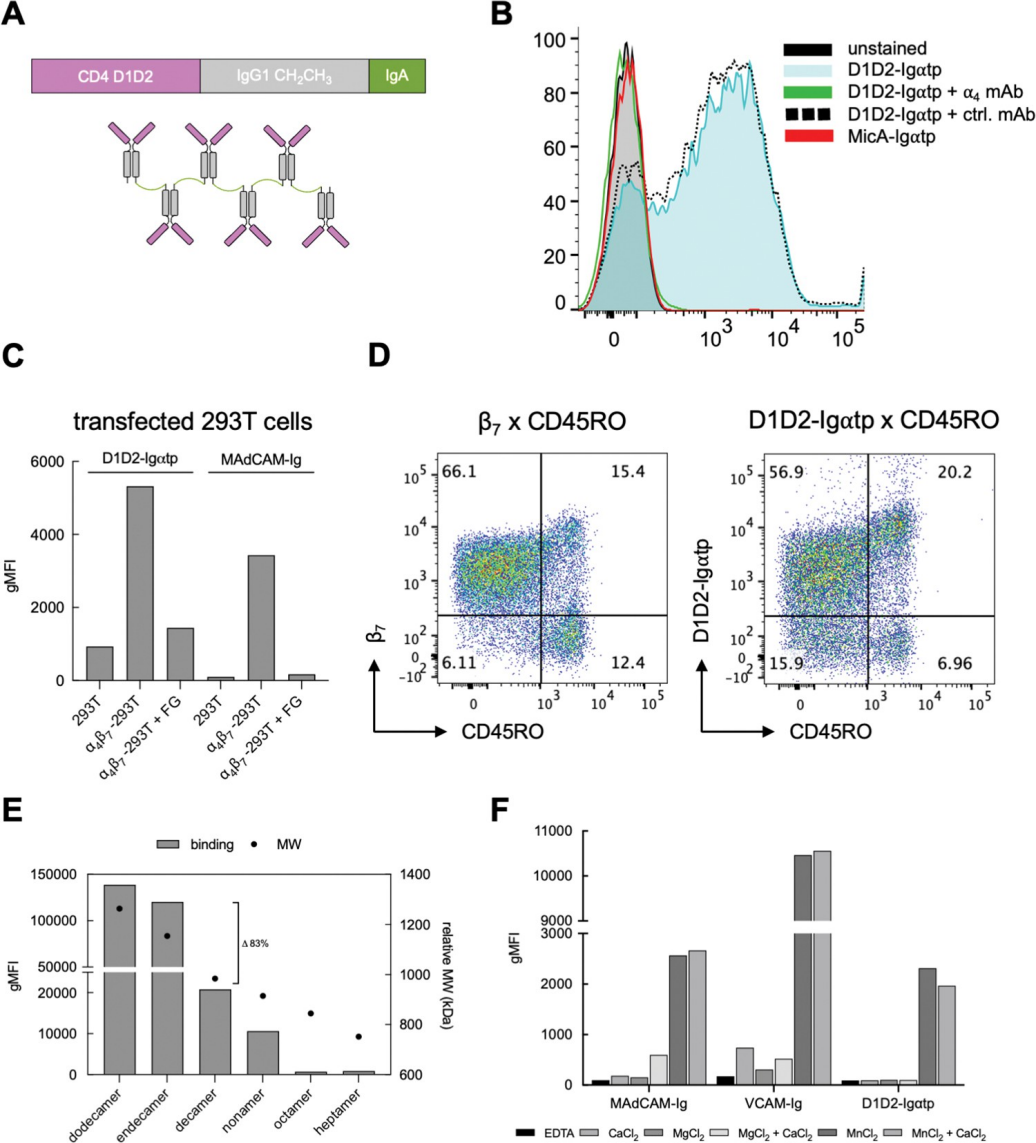
Binding of soluble CD4 to ⍺_4_β_7_ on CD4^+^ T cells. (A) Schematic of D1D2-Ig⍺tp. (B) Flow-cytometry histogram of PE-conjugated D1D2-Ig⍺tp staining of CD4^+^ T cells alone (blue) or in the presence of either the anti ⍺_4_ mAb 2B4 (green) or an irrelevant mAb (black-dashed). Cells stained with PE-conjugated MicA-Ig⍺tp (red), along with unstained cells (grey) are included. X-axis indicates geometric mean fluorescence intensity (gMFI). (C) 293T cells untransfected or co-transfected with ⍺_4_ and β_7_ expression vectors stained with either PE-D1D2-Ig⍺tp or PE-MAdCAM-Ig in the absence or presence of the ⍺_4_ antagonist FG. Y-axis indicates gMFI. (D) CD4^+^ T cells stained with anti CD45RO (x-axis) anti β_7_ (y-axis) (left), or with anti CD45RO (x-axis) and D1D2-Ig⍺tp (y-axis) (right). % cells in each quadrant, as indicated. (E) CD4^+^ T cells stained as in B, with lower MW forms of oligomerized D1D2-Ig⍺tp. Left Y-axis indicates gMFI (bars), right Y-axis indicates relative MW (kDa) (•). (F) Staining of primary CD4^+^ T cells with PE-conjugated D1D2-Ig⍺tp, PE-MAdCAM-Ig or PE-VCAM-Ig, as in panel B in the presence of buffers containing different combinations of CaCl_2_, MgCl_2_ and MnCl_2_. Y-axis indicates gMFI.

The FRET/FLIM results described above indicate a direct interaction between CD4 and ⍺_4_β_7_. A determination that CD4 exhibits a specific affinity for ⍺_4_β_7_ would support this finding. To this end we employed a fluorescently labelled soluble dodecameric CD4 Ig fusion protein, termed D1D2-Ig⍺tp, that includes the two N-terminal Ig-like domains of CD4 (D1D2, aa 1–180), followed by IgG1 CH2 and CH3 domains, followed by the 18 aa tail-piece domain of IgA (⍺tp) [45]. In this oligomer, 12 D1D2-Igs are arranged in a disulphide linked daisy-chain (Fig 1A). Primary CD4^+^ T cells, some of which express ⍺_4_β_7_, were reacted with D1D2-Ig⍺tp and analyzed by flow-cytometry. A second oligomeric Ig fusion protein, MicA-Ig⍺tp, which includes the two N-terminal domains of MicA, was employed as a specificity control. D1D2-Ig⍺tp, but not MicA-Ig⍺tp, bound to cells, and this binding was inhibited by an integrin ⍺_4_ mAb but not by a control mAb (Fig 1B). To establish specificity, 293T cells were co-transfected with ⍺_4_ and β_7_ expression vectors and stained with D1D2-Ig⍺tp in the absence or presence of the 499.5 Da ⍺_4_ integrin antagonist firategrast (FG) [46]. MAdCAM-Ig was employed as a positive control. Both D1D2-Ig⍺tp and MAdCAM-Ig bound preferentially to ⍺_4_β_7_ transfected cells, and in both cases, binding was inhibited by FG (Fig 1C). We conclude that an oligomeric soluble CD4 that includes only its two N-terminal domains can bind to ⍺_4_β_7_ in a specific way.

⍺_4_β_7_ is expressed at intermediate levels on the majority of naïve CD4^+^ T cells in blood. As noted above, most memory CD4^+^ T cells in blood show either little or no ⍺_4_β_7_ expression. However a minor subset (~5–20%) express high levels of ⍺_4_β_7_. Differential expression of ⍺_4_β_7_ on naïve vs memory cell can be visualized by co-staining cells with β_7_ and CD45RO mAbs (Fig 1D). We determined that D1D2-Ig⍺tp bound, like β_7_ mAb, to a minor subset of memory cells at relatively high levels, and to naïve cells at a lower level. Although we do not exclude the possibility of trans interactions, the direct interaction we show by FRET/FLIM, combined with the specific binding of CD4 to ⍺_4_β_7_ leads us to conclude that CD4 and ⍺_4_β_7_ interact in cis on the surface of CD4^+^ T cells (Figs 1 and S1). Molecular details of CD4-⍺_4_β_7_ interactions, described below, support this conclusion.

We next compared the ⍺_4_β_7_ reactivity of dodecameric D1D2-Ig⍺tp to smaller oligomers. While endecamers and decamers bound ⍺_4_β_7_ at levels comparable to dodecamers, we observed a marked reduction (~80%) in binding with smaller forms (Fig 1E). We note that for many integrins, including ⍺_4_ integrins, ligand recognition is modulated by clustering and avidity effects [47]. Finding that only higher order CD4 oligomers bind ⍺_4_β_7_ is consistent with the role that avidity frequently plays in integrin-ligand interactions. Moreover, it helps explain how the specific affinity of CD4 for ⍺_4_β_7_ has gone unrecognized until now.

MAdCAM-1 and VCAM-1 bind to the N-terminal headpiece of ⍺_4_β_7_ only when it adopts an active conformation [48]. ⍺_4_β_7_ conformation is regulated via “inside out signaling” [49], but can be artificially manipulated in vitro by altering relative concentrations of divalent cations. High Mn^++^ concentrations promote an active state. We compared the conformational requirements of CD4 binding to ⍺_4_β_7_ with those of MAdCAM-1 and VCAM-1 by staining ⍺_4_β_7_ -expressing RPMI8866 cells with D1D2-Ig⍺tp in 6 buffers supplemented with different combinations of divalent cations. Like MAdCAM-Ig and VCAM-Ig, D1D2-Ig⍺tp failed to bind ⍺_4_β_7_ in the absence of divalent cations (Fig 1F). All three ligands bound to ⍺_4_β_7_ in the presence of Mn^++^. We observed low levels of binding of the two natural ligands in the presence of Mg^++^ and no binding of D1D2-Ig⍺tp. We conclude that D1D2-Ig⍺tp selectively engages an activated form of ⍺_4_β_7_ normally associated with ligand binding.

### MAdCAM-1 and VCAM-1 inhibition of CD4 binding to ⍺_4_β_7_

**Fig 2:**
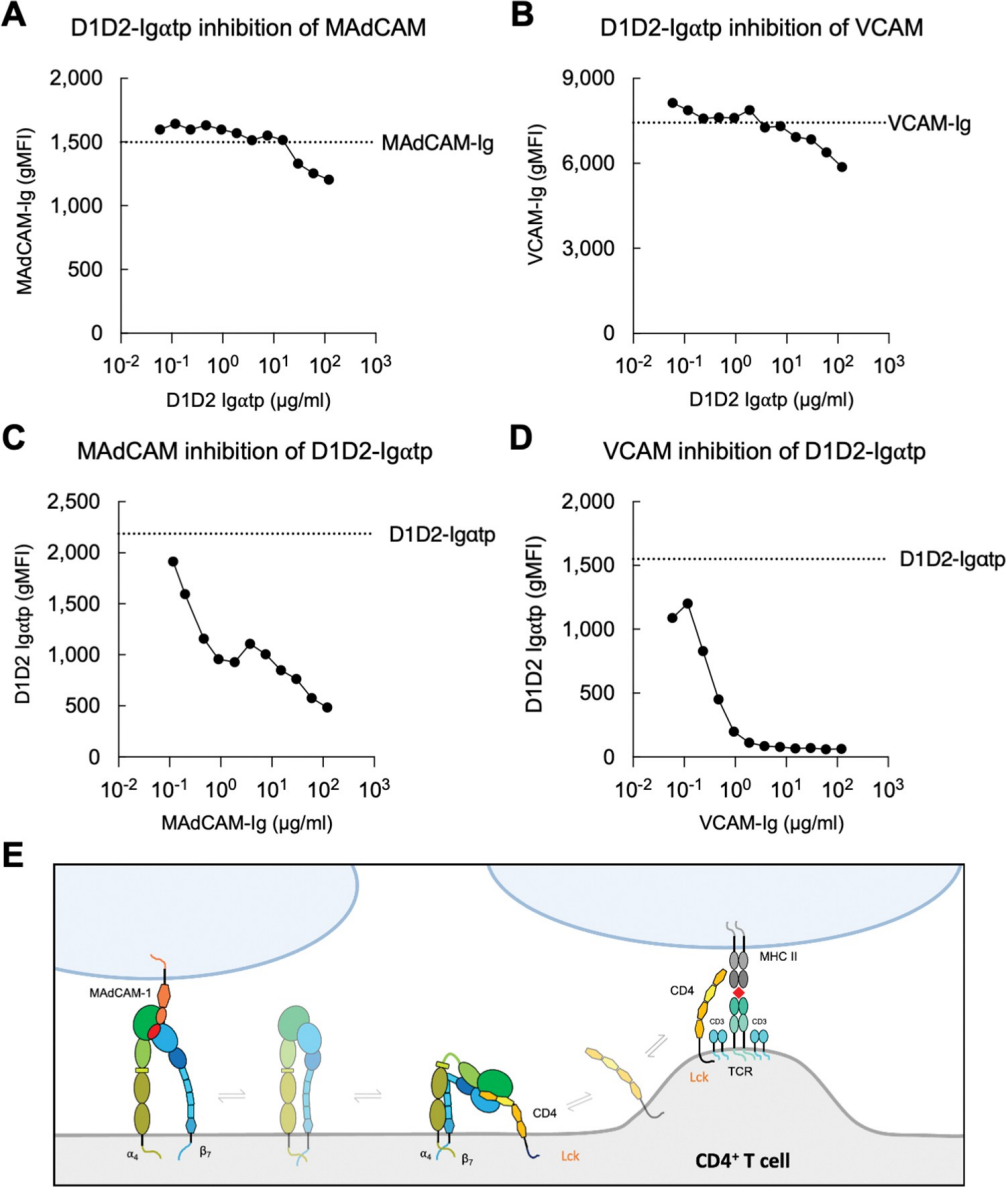
Cross-inhibition of MAdCAM-Ig, VCAM-Ig and D1D2-Ig⍺tp binding to ⍺_4_β_7_. Flow cytometric analysis of (A) PE -conjugated MAdCAM-Ig or (B) PE -conjugated VCAM-Ig binding to RPMI8866 cells pre-incubated with increasing concentrations of unlabeled D1D2-Igαtp. Cell preincubated with increasing concentrations of (C) unlabeled MAdCAM-Ig or (D) VCAM-Ig followed by PE -conjugated D1D2-Ig⍺tp. Y-axis indicates gMFI. Dashed line indicates gMFI in the absence of any inhibitor. (E) Schematic of a cis interaction between CD4 D1D2 and the headpiece domain of α_4_β_7_ can potentially inhibit α_4_β_7_ trans interactions with MAdCAM (left), or CD4 migration into the TCR and trans interactions with MHC II (right).

Cis interactions between various integrins and leukocyte receptors are known to regulate immune cell functions [37,40–42]. To determine whether CD4 inhibits MAdCAM-1 or VCAM-1 binding to ⍺_4_β_7_ we preincubated RPMI8866 cells with labelled MAdCAM-Ig or VCAM-Ig in the presence of increasing concentrations of unlabeled D1D2-Ig⍺tp. We observed only minor inhibition at high D1D2-Ig⍺tp concentrations (Fig 2A and 2B). In contrast both MAdCAM-Ig and VCAM-Ig inhibited D1D2-Ig⍺tp binding to ⍺_4_β_7_ in a dose dependent manner (Fig 2C and 2D). This implies that MAdCAM-1 and VCAM-1, acting in trans, may interfere with CD4-⍺_4_β_7_ interactions. What remains unknown is whether interactions between ⍺_4_β_7_ and CD4 impact CD4 migration into the TCR and subsequent interactions with MHC II (Fig 2E).

### Therapeutics developed to treat inflammatory diseases inhibit CD4 - ⍺_4_β_7_ interactions

**Fig 3:**
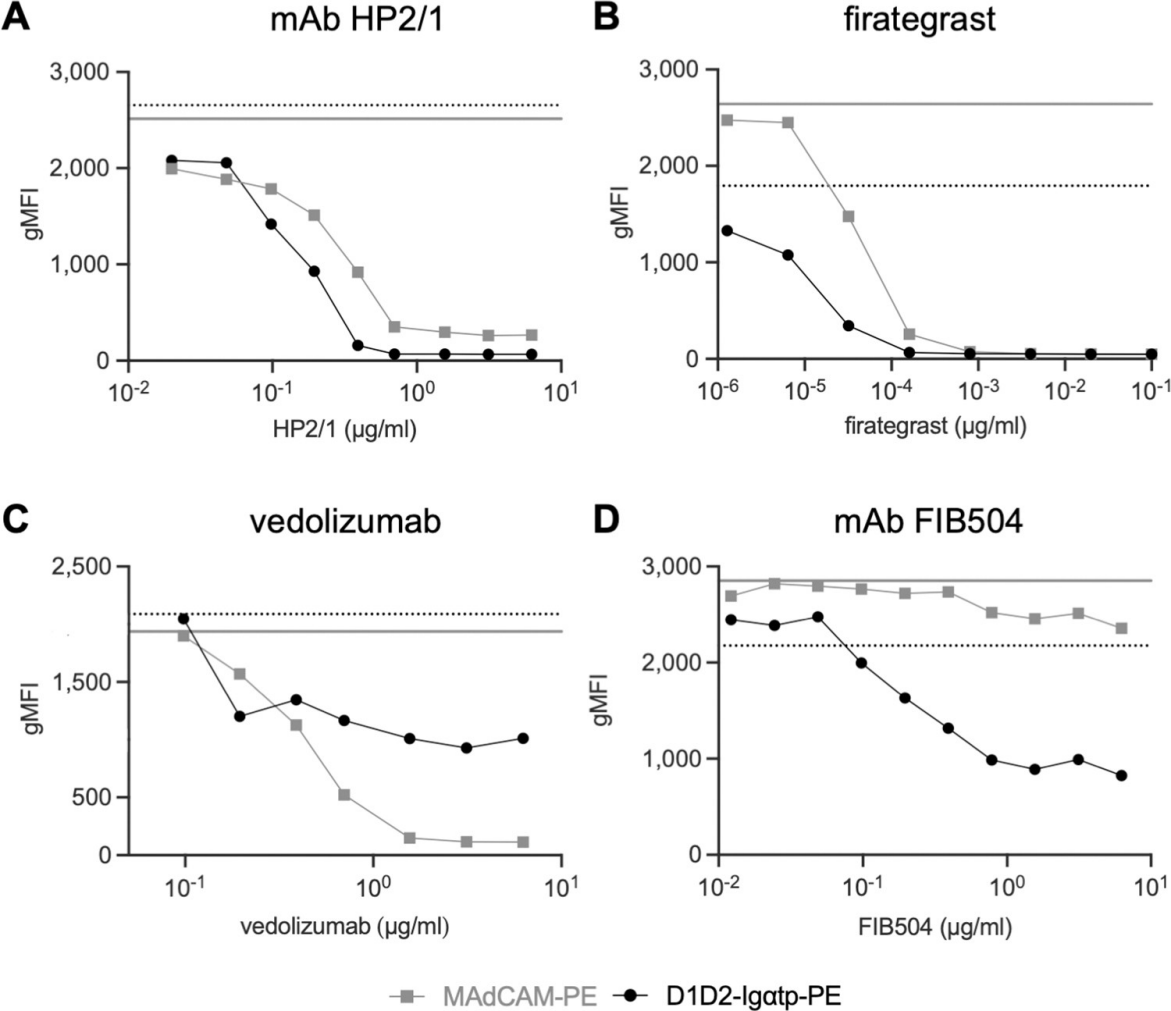
Inhibition of D1D2-Ig⍺tp and MAdCAM-Ig by ⍺_4_ and ⍺_4_β_7_ mAb antagonists. Flow-cytometric analysis of ⍺_4_β_7_^+^ cells stained with PE-conjugated MAdCAM-Ig (grey) or D1D2-Ig⍺tp (black) in the presence of increasing concentrations of (A) ⍺_4_ mAb HP2/1, (B) firategrast, (C) vedolizumab or (D) β_7_ mAb FIB504. Y-axis indicates gMFI. Dashed and solid lines represent gMFI of D1D2-Ig⍺tp and MAdCAM-Ig respectively, in the absence of any antagonist.

Several mAbs and small molecule antagonists of ⍺_4_β_7_ and ⍺_4_β_1_ have been developed to treat diseases, including IBD and MS. Although their specificities vary, they all inhibit ⍺_4_β_7_ binding to MAdCAM-1. Vedolizumab and etrolizumab are β_7_ mAbs, based on murine mAbs Act-1 and FIB504, respectively. Both are used to treat IBDs, [50,51]. Natulizumab is an ⍺_4_ specific humanized version of the mouse mAb HP2/1 used to treat Multiple Sclerosis (MS) [52]. FG is a small-molecule MAdCAM-1 and VCAM-1 inhibitor developed to treat MS [46]. To determine whether these antagonists block CD4 binding to α_4_β_7_, we stained RPMI8866 cells with D1D2-Ig⍺tp in the absence or presence of increasing concentrations of each (Fig 3). HP2/1 inhibited both D1D2-Ig⍺tp and MAdCAM-Ig to near completion at a concentration of 1μg/ml. FG also inhibited both ligands. Vedolizumab blocked MAdCAM-Ig efficiently at 1 μg/ml but was only able to reduce D1D2-Ig⍺tp binding ~50% at a concentration of 10 μg/ml. FIB504 partially inhibited (~60% at 10 μg/ml) D1D2-Ig⍺tp binding to ⍺_4_β_7_. In sum, although their patterns of inhibition varied, each of these ⍺_4_ and β_7_ antagonists interfered with CD4-α_4_β_7_ interactions.

### Peptide scanning of the ⍺_4_β_7_ binding site(s) on CD4 D1D2

To measure the specific affinity of CD4–⍺_4_β_7_ interactions we developed a surface plasmon resonance (SPR) -based assay in which recombinant ⍺_4_β_7_ was passed over a surface coated with a 23 kD D1D2 protein (aa 1–183) [8]. For comparison we prepared surfaces with recombinant MAdCAM-Ig, VCAM-Ig, monomeric gp120, and a SOSIP env trimer (S1 Table). Assays were carried out in the presence of MnCl_2_ to present ⍺_4_β_7_ in an active conformation. We obtained a dissociation constant (KD (M)) between ⍺_4_β_7_ and CD4 of 3.57e^-9^. This rate of dissociation was similar to that of both MAdCAM-Ig and VCAM-Ig with ⍺_4_β_7_ and is indicative of a relatively high-affinity interaction.

**Fig 4:**
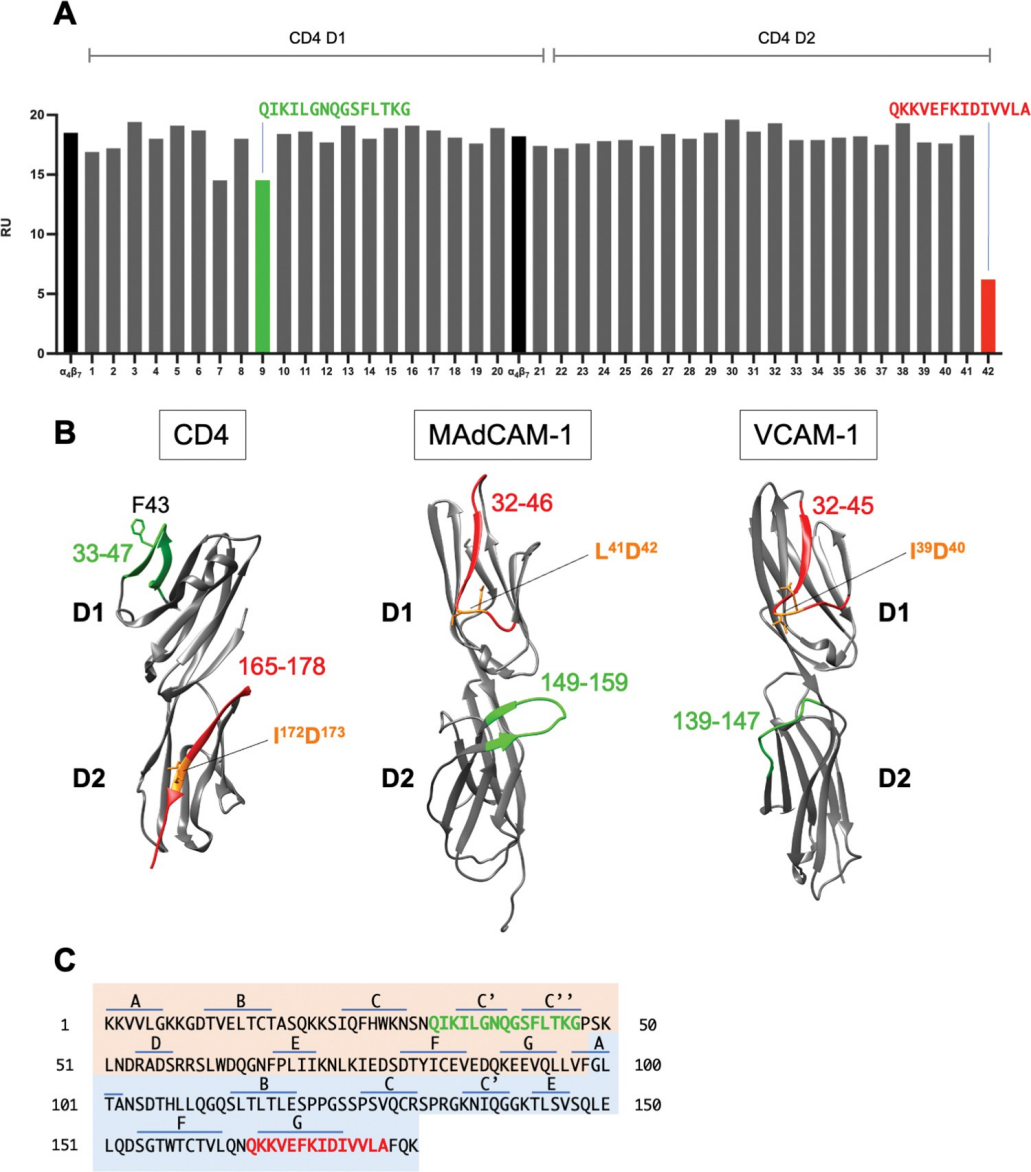
CD4 peptide inhibition of soluble ⍺_4_β_7_ binding to CD4 D1D2. (A) SPR assay of soluble ⍺_4_β_7_ binding to a biosensor surface coated with sCD4 D1D2 in the absence (black) or presence of overlapping 15 aa peptides corresponding to residues encoded in CD4 D1 (p1-p21) and D2 (p22-p42). Binding of ⍺_4_β_7_ to the D1D2 coated surface is reported in response units (RU). p9 (green) and p42 (red) aa sequences are reported. (B) Ribbon diagram of D1 and D2 domains of CD4 (PDB: 1CDI), MAdCAM-1 (PDB: 1BQS) and VCAM-1 (PDB: 1IJ9). 1° binding sites are highlighted in red, and accessory binding sites are highlighted in green. Predicted metal ion coordination sites for each ligand are indicated (orange). (C) CD4 D1D2 residues with β-strand designations listed above. D1 residues are highlighted in orange and D2 residues in blue. p9 and p42 are highlighted in red and green.

We next modified our SPR -based assay in order to map the site(s) on CD4 that interacts with ⍺_4_β_7_. In this modified assay ⍺_4_β_7_ was passed over a surface coated with CD4 D1D2 in the absence or presence of 42 overlapping 15 amino acid linear peptides spanning CD4 residues 1–178 (S2 Table). Two peptides located in CD4 D1, #7 and #9, inhibited ⍺_4_β_7_ binding by ~17% (Fig 4A). The final peptide in CD4 D2, p42, inhibited binding by ~ 66%. Linear peptides often lack the true conformation of the protein from which they are derived. Additionally, the kinetic format of our assay allows for ligand-analyte rebinding during the association phase. These features render this inhibition assay somewhat insensitive. Nevertheless, the dynamic range of this assay is sufficient to identify interactions that are worthy of follow-up investigation (S4 Fig). As such, our preliminary findings raised the possibility that both D1 and D2 domains of CD4 interact with ⍺_4_β_7_. Of note, MAdCAM-1 and VCAM-1, like CD4, present two tandemly arrayed N-terminal IgSF domains (Fig 4B). In both cases, binding to ⍺_4_β_7_ is mediated by a 1° binding site in their D1s and an accessory site, located in their D2s. The term accessory is a misnomer insofar as these sites plays an essential role in ⍺_4_β_7_ binding. Follow-up experiments focused on p9 and p42, which we herein refer to as pCD4^33-47^ and pCD4^165-178^. pCD4^33-47^ partially overlaps the D1 C’C” β-strands and a connecting loop. The primary gp120 contact site (Phe^43^) lies at the N-terminal edge of this loop (Fig 4C). pCD4^165-178^ includes Q^165^ and K^166^. These residues lie primarily within the G β-strand of D2, close to the boundary with D3. Because peptide inhibition assays sometime mediate nonspecific effects, further analysis was performed to better establish the role of these two regions of CD4 in binding to ⍺_4_β_7_.

### CD4^165-178^ binds directly to ⍺_4_β_7_

**Fig 5:**
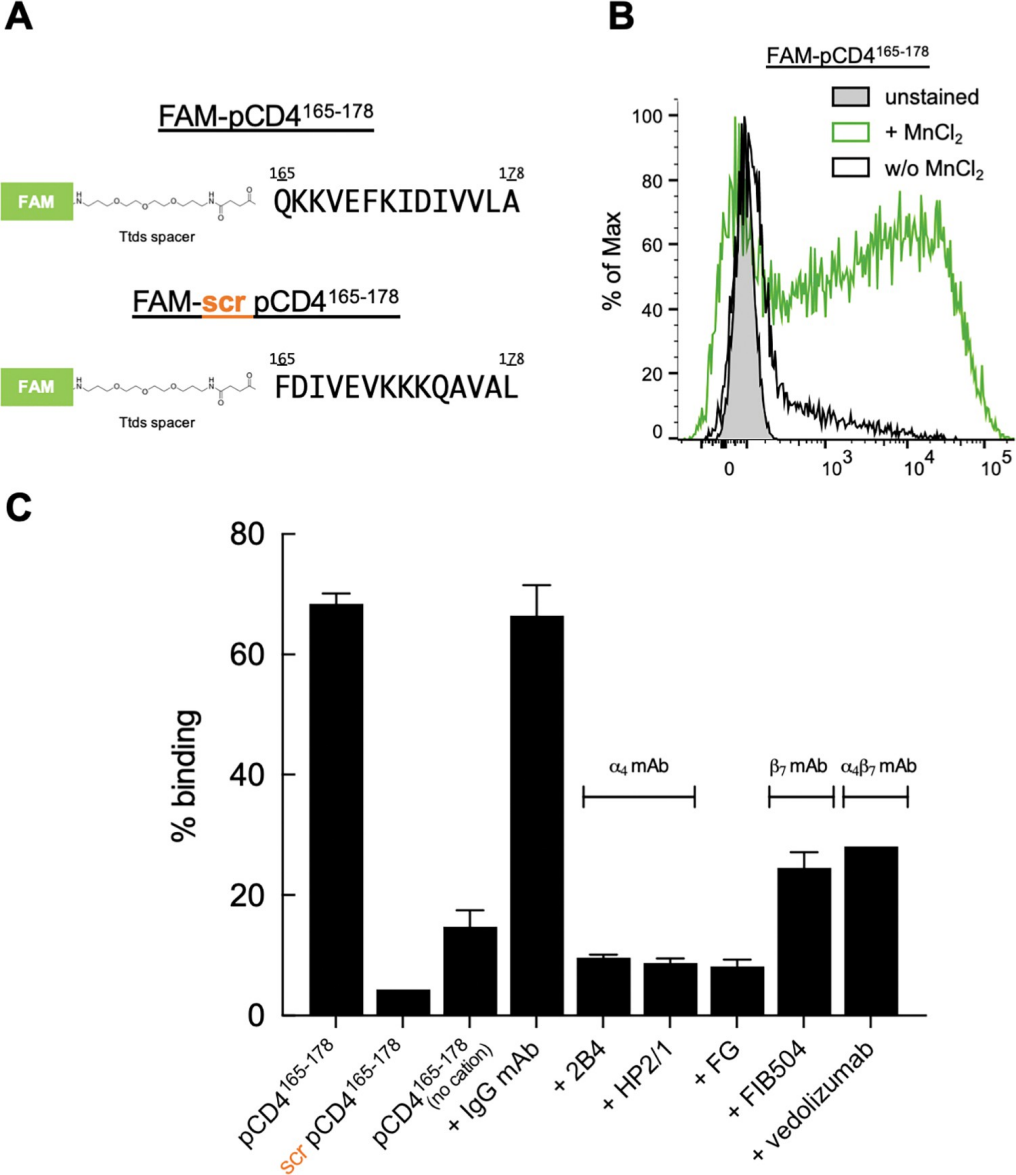
Direct binding of pCD4^165-178^ to ⍺_4_β_7_ on primary T cells. (A) Sequence of FAM -conjugated pCD4^165-178^ and a scrambled derivative (FAM-scr pCD4^165-178^). (B) Flow-cytometry derived histogram of pCD4^165-178^ binding to RA -cultured CD8^+^ T cells. pCD4^165-178^ in the presence (green), or absence of Mn^++^(black). Unstained cells are shown (grey). (C) Binding of pCD4^165-178^ and its scrambled derivative to CD8^+^ T cells in the presence of MnCl_2_ as in panel B, and in the presence of ⍺_4_ and ⍺_4_β_7_ antagonists, as indicated. Y-axis indicates % cells bound. Error bars represent standard error from 3 independent experiments.

To further investigate the interaction between pCD4^165-178^ and ⍺_4_β_7_ we attached a fluorescein dye to its amino terminus via a trioxatridecan-succinamic acid (Ttds) linker (Fig 5A). A scrambled pCD4^165-178^ was employed as a control. Both peptides were reacted with RA -treated primary CD8^+^ T cells that express high levels of ⍺_4_β_7_. pCD4^165-178^ reacted strongly in the presence, but not in the absence of Mn^++^ (Fig 5B). The scrambled pCD4^165-178^ showed limited binding (Fig 5C). ⍺_4_ mAbs 2B4 and HP2/1 reduced binding by ~ 80%, as did FG. FIB504 and vedolizumab inhibited binding by ~50%. This inhibition pattern resembles the one we obtained when inhibiting D1D2-Ig⍺tp with the same antagonists (Fig 3). We conclude that the CD4 D2 G β-strand binds directly to the headpiece region of ⍺_4_β_7_ in a cation dependent manner. Support for this conclusion, in the form of site directed mutagenesis within the D2 G β-strand is provided below.

### CD4^165-178^ shares a sequence motif with gp120 V2

**Fig 6:**
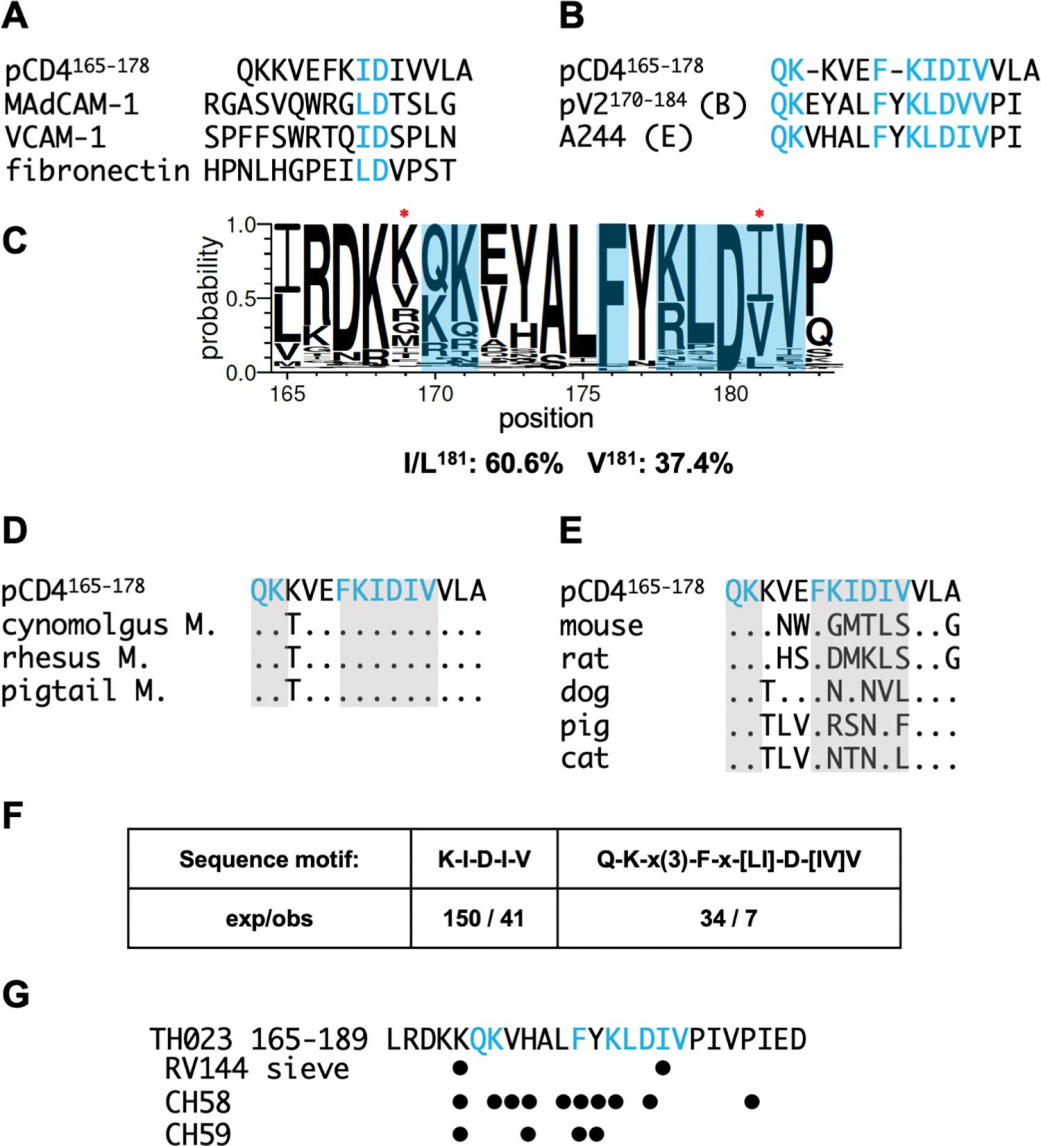
A shared sequence motif in gp120 V2 and the CD4 D2. (A) Alignment of the ⍺_4_β_7_ 1° binding site in MAdCAM-1 D1 (aa 32–46), VCAM-1 D1 (aa 32–45) and CD4 D2 (aa 165–178). I/LD metal ion coordination sites are highlighted in blue. (B) Alignment of the 1° ⍺_4_β_7_ binding site in CD4 D2 with the V2 mid-region (aa 170–184) in a consensus B and A244 (subtype E) gp120. Aligned sequences are highlighted in blue, and spaces are represented by a dash. (C) Global sequence variation plot of gp120 V2 aa 165–183. Relative amino acid representation among sequences deposited in the LANL database is proportional to font size. Red asterisks indicate sieve residues in RV144 infected subjects. Frequency of I^181^ or L^181^ vs V^181^ is noted. (D) Alignment of CD4 D2 aa 165–178 with corresponding CD4 D2 residues from NHPs. Dots indicate sequence identity. (E) Expected and observed matches of a short and extended version of the 1° ⍺_4_β_7_ binding site motif in CD4 D2 found in the Swiss-Prot protein database. (F) 92TH023 gp120 aa 165–189, with aa relevant to mAbs CH58 and CH59 reactivity indicated (•).

MAdCAM-1, VCAM-1 and fibronectin each encode an essential aliphatic aa-Asp motif in their 1° ligand binding site (MAdCAM-1: LD, VCAM-1: ID, fibronectin: LD). The Asp coordinates with a Mg^++^ ion that sits in an ⍺_4_β_7_ metal ion -dependent adhesion site (MIDAS). pCD4^165-178^ includes such a motif: I^172^D^173^, consistent with its cation -dependent binding to α_4_β_7_. Using I^172^ and D^173^ as anchor residues we aligned pCD4^165-178^ to the MAdCAM-1 and VCAM-1 1° ligand binding sites [53,54], but found no additional sequence similarity (Fig 6A).

We and others have previously reported that some recombinant gp120s bind to ⍺_4_β_7_ in a cation -dependent manner [26–28,55,56] and determined that this interaction is mediated by conserved residues in the V2 domain. In support of this finding, a subtype B V2 consensus peptide spanning residues 170–184 (pV2^170-184^) inhibits gp120 binding to ⍺_4_β_7_ [56]. pV2^170-184^ includes a conserved aliphatic aa-Asp motif (L^179^D^180^). Substitution of L^179^D^180^ reduces gp120 binding to ⍺_4_β_7_ substantially [26]. Cardozo and colleagues showed, using a similar V2 peptide, that aa 170–172 (consensus QK[VE]) also plays a key role in gp120 binding to α_4_β_7_ [28]. With these observations in mind, we aligned pCD4^165-178^ with pV2^170-184^ (Fig 6B). Sequence similarity was evident. Most striking, CD4 residues 171–175 (KIDIV) are similar to V2 residues 178–182 (KLDVV). In V2, K^178^, L^179^ and D^180^ are the most frequently encoded amino acids at their respective positions (Fig 6C). Although V^181^ in our subtype B V2 peptide does not align with CD4 I^174^, the presence of valine at this position is atypical. The majority of gp120s (61%) in the Los Alamos National Laboratory (LANL) database encode either an isoleucine or leucine at position 181, such that the CD4 D2 sequence KIDIV is nearly identical to KLDIV, the most common V2 sequence spanning aa 178–182, and both include a metal ion coordination motif (ID and LD). Three additional conserved residues in this region of V2, Q^170^, K^171^, and F^176^ could also be aligned with residues in pCD4^165-178^. In sum, a sequence overlapping the G β-strand of CD4 D2, that binds directly to ⍺_4_β_7_, is nearly identical to a conserved sequence in gp120 V2 that also binds to ⍺_4_β_7_.

Inspection of nonhuman primate orthologs indicates that CD4 residues Q^165^, K^166^, F^170^, along with KIDIV (aa 171–175) are conserved in 19 of 21 Old World monkeys, including those of Asian origin that are employed in NHP models of HIV infection, but are absent in New World monkeys and other mammals (Figs 6D, 6E and S3).

Next, we considered the possibility that similar sequences might appear frequently in other cell surface proteins, particularly proteins with Ig -like domains, which would reduce the significance of the sequence similarity we observed. To this end, we queried the ~ 560,000 proteins included in the SwissProt database. We initially analyzed a motif, QKx(3)Fx[LI]D[IV]V, that includes all of the residues in CD4 D2 that align with gp120 V2 (x is any amino acid and brackets allow for either of two residues at a single position). Using the ScanProsite tool we determined that the probability this motif would appear randomly was 6.8 per 100,000 (p = 6.8e^-6^) (Fig 6F). We obtained 7 hits, all of which were primate CD4 genes (S3 Table). We then restricted our search to include only KIDIV. The probability that this sequence appears randomly is 30 per 100,000 (p = 3e^-5^). We observed 41hits among 560,000 proteins. 8 were primate CD4 genes. The only other cell surface receptor encoding KIDIV was IL-1R, which does not react with ⍺_4_β_7_. This suggests that other elements of CD4 D2 are necessary for ⍺_4_β_7_ reactivity. These findings indicate that a CD4 D2 sequence that binds directly to ⍺_4_β_7_ is not common in the eukaryotic proteome, indicating that the presence of a closely related sequence in the gp120 V2 domain was worthy of further investigation.

### CD4^165-178^ and V2^170-184^ cross-inhibit ⍺_4_β_7_ ligands

**Fig 7:**
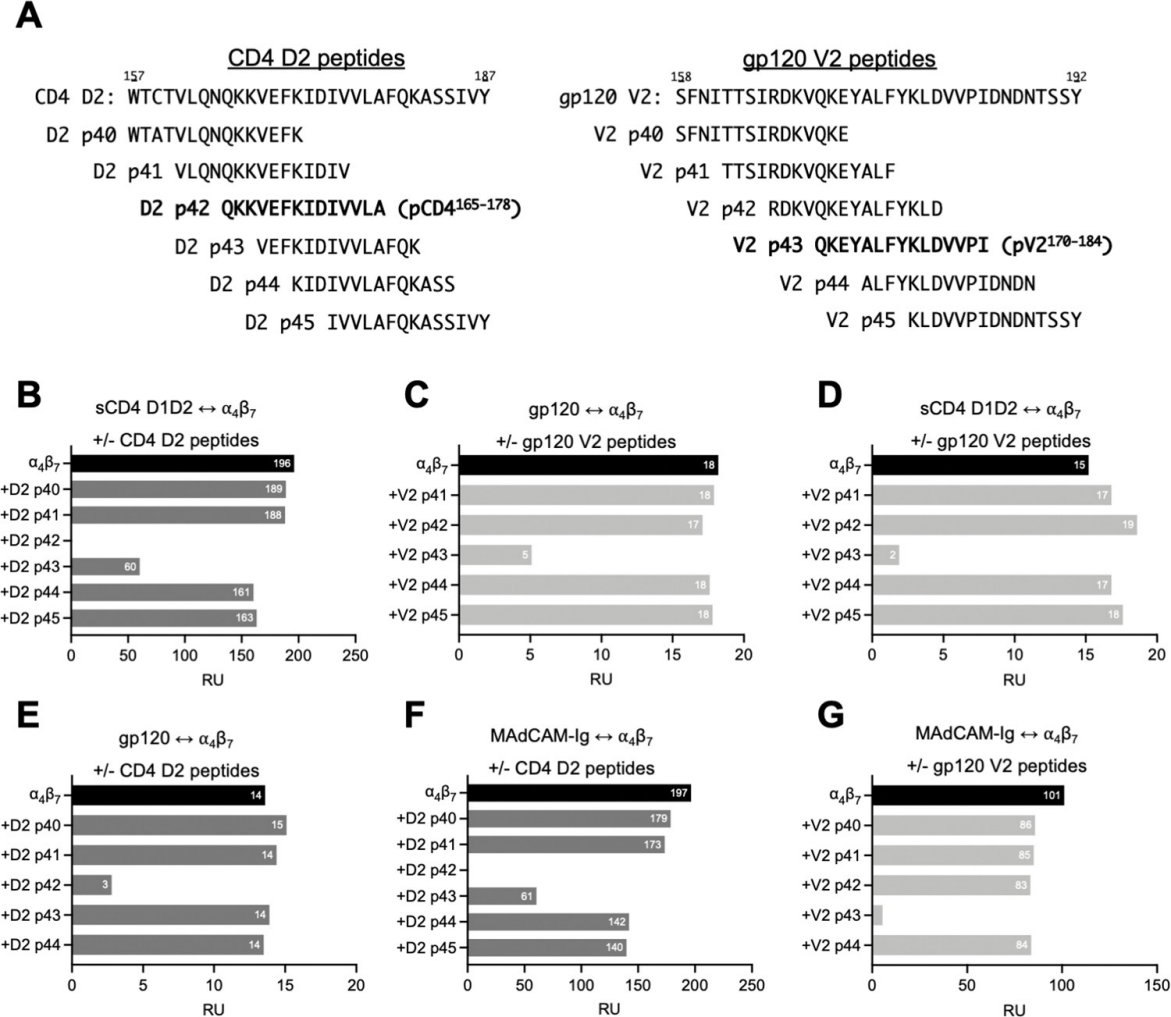
Reciprocal inhibition of gp120 V2 and CD4 binding to ⍺_4_β_7_ by D2 and V2 peptides. SPR measurements of soluble ⍺_4_β_7_ (analyte) binding to biosensor surfaces coated with CD4 D1D2, 92TH023 cyclic peptide, or MAdCAM-Ig in the absence or presence of either CD4 D2 or gp120 V2 peptides, as indicated. (A) List of overlapping 15 aa peptides corresponding to residues 157–187 of CD4 and residues 158–192 of gp120 V2 (subtype B consensus sequence). (B) Binding of ⍺_4_β_7_ to sCD4 D1D2 +/- CD4 D2 peptides. X-axis indicates ⍺_4_β_7_ mass bound (RU) at the end of the association phase. (C) Binding of ⍺_4_β_7_ to 92TH023 +/- gp120 V2 peptides. (D) Binding of ⍺_4_β_7_ to sCD4 D1D2 +/- gp120 V2 peptides. (E) Binding of ⍺_4_β_7_ to 92TH023 +/- CD4 D2 peptides. (F) Binding of ⍺_4_β_7_ to MAdCAM-Ig +/- CD4 D2 peptides. (G) Binding of ⍺_4_β_7_ to MAdCAM-Ig +/- gp120 V2 peptides.

The sequence similarity between pCD4^165-178^ and pV2^170-184^ suggested that they engage the ⍺_4_β_7_ headpiece in a similar way. To determine if this is the case, we employed an SPR -based assay. Biosensor surfaces were coated with either CD4 D1D2 or a 42 aa cyclic V2 peptide derived from the subtype E 92TH023 gp120 (cV2 92TH023). A third surface coated with MAdCAM-Ig was employed as a positive control. Soluble ⍺_4_β_7_ was passed over these surfaces. We then evaluated the capacity of staggered 15 aa peptides that span either CD4 D2 or gp120 V2 to inhibit this interaction (Fig 7A).

Before proceeding, we evaluated CD4 peptides p43, p44 and p45 that extend our initial screen presented in Fig 4A to include residues in D2 c-terminal to A^178^, the final residue in pCD4^165-178^. p44 and p45 were unable to inhibit ⍺_4_β_7_ binding to D1D2 (Fig 7B), indicating that CD4 residues c-terminal to A^178^ do not likely contribute to ⍺_4_β_7_ reactivity. p43 showed reduced inhibition relative to pCD4^165-178^, suggesting that Q^165^ and K^166^, which are absent in p43 but present in pCD4^165-178^, contribute to the ⍺_4_β_7_ reactivity. This is notable because these two residues align with gp120 V2 aa Q^170^K^171^, which were singled out by Cardozo and colleagues as key determinants of V2-⍺_4_β_7_ reactivity [28].

We previously reported that pV2^170-184^ inhibits A244 gp120 binding to ⍺_4_β_7_ [56], and reproduced that result here for reference (Fig 7C). We then determined that pV2^170-184^ inhibits D1D2 binding to ⍺_4_β_7_ (Fig 7D), and pCD4^165-178^ inhibits gp120 binding to ⍺_4_β_7_ (Fig 7E). Finally, both pCD4^165-178^ and pV2^170-184^ inhibit MAdCAM-Ig binding to ⍺_4_β_7_ (Fig 7F and 7G). In summary, pCD4^165-178^ and pV2^170-184^ interchangeably inhibit gp120, CD4 D1D2, and MAdCAM-Ig binding to ⍺_4_β_7_. Considering that CD4 is relatively invariant, and gp120 V2 is under continuous selection, we conclude that gp120 V2 mimics the G β-strand of CD4 D2.

As presented in S3 Fig the G β-strand of CD4 D2 is well conserved in Old World monkeys, but not in New World monkeys. Because divergent sequences can adopt similar structures, we asked whether a D2 G β-strand peptide derived from a New World monkey CD4 could retain a structure required to bind ⍺_4_β_7_. *Aotus nancymaae* is a South American night monkey whose D2 G β-strand sequence diverges from human CD4 in a significant way. An Aotus peptide analogous to pCD4^165-178^ was employed as an inhibitor of ⍺_4_β_7_ binding to surface immobilized D1D2, as described above. We included a Gorilla pCD4^165-178^, which differs slightly from human CD4, as a reference. The Aotus and Gorilla peptides both inhibited ⍺_4_β_7_ binding in a similar way (S5 Fig). This suggests that the cis interaction between ⍺_4_β_7_ and CD4 is retained in Aotus, and perhaps other New World monkeys and demonstrates that ⍺_4_β_7_ can retain CD4 binding even with a sequence that diverges significantly from the conserved KIDIV sequence. This observation is consistent with gp120 V2 mimicking CD4 when one considers that HIV evolved out of Old World monkeys.

### A gp120 binding residue in CD4 D1 mediates ⍺_4_β_7_ -reactivity

**Fig 8:**
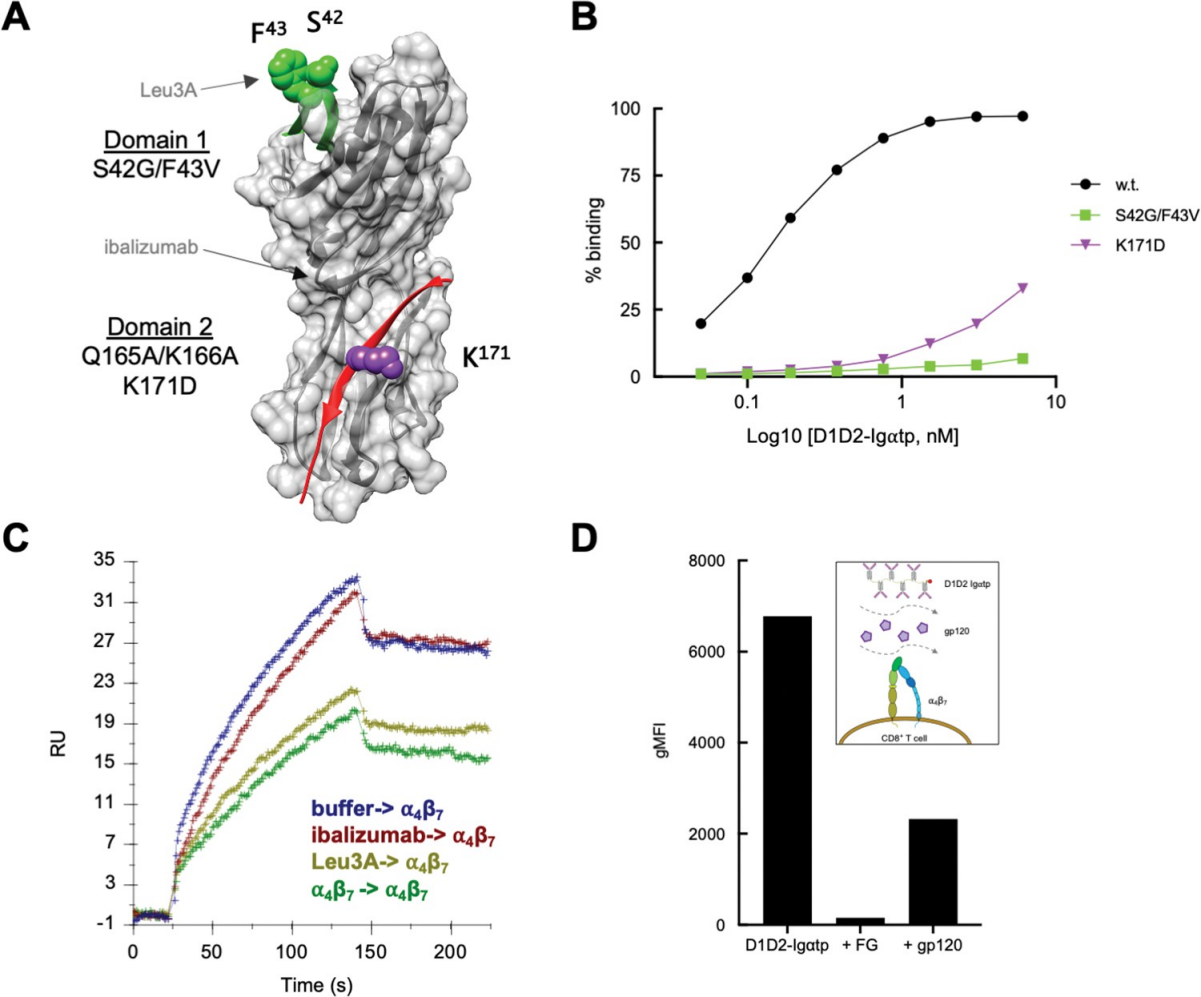
Mutating a gp120 contact site in CD4 D1 abrogates CD4-⍺_4_β_7_ interactions. (A) Positions of amino acid substitutions introduced into D1D2-Ig⍺tp are indicated on a space-filling model of CD4 D1D2. Substitutions at S42/F43 in D1 (green) and K171 in D2 (purple) are shown. Approximate locations of mAb Leu3A and ibalizumab epitopes are indicated. (B) Flow-cytometric analysis of increasing amounts of D1D2-Ig⍺tp and mutant derivatives binding to ⍺_4_β_7_ on CD8^+^ T cells. Y-axis indicates % cells bound. (C) SPR analysis of soluble ⍺_4_β_7_ binding to a D1D2 coated surface following preincubation of the surface with ibalizumab (red), mAb Leu3A (light green), or ⍺_4_β_7_ (dark green). Y-axis indicates the mass of soluble ⍺_4_β_7_ bound at the end of the association phase. Mass of prebound inhibitors is subtracted. (D) Flow-cytometric analysis of unlabeled gp120 inhibiting PE labeled D1D2-Ig⍺tp binding to ⍺_4_β_7_ on CD8^+^ T cells. The integrin ⍺_4_ antagonist FG was employed as a control. Y-axis indicates MFI.

Both MAdCAM-1 and VCAM-1 utilize two binding sites (1° and accessory) to engage ⍺_4_β_7_. Our preliminary peptide scan (Fig 4) indicated that pCD4^33-47^, which overlaps a loop connecting the C’-C” strands in D1, inhibited ⍺_4_β_7_ binding in a modest way (~17%). This observation, by itself, is insufficient to conclude that CD4 D1 is involved in binding to ⍺_4_β_7_. However, with MAdCAM-1 and VCAM-1 in mind, we explored this possibility further. MAdCAM-1 and VCAM-1 accessory sites, which are both loops, were initially identified by site directed mutagenesis [53,54]. Mutating these accessory sites reduced their ⍺_4_β_7_ reactivity dramatically. We followed a similar strategy for CD4 by constructing S42G/F43V-D1D2 -Igαtp, which incorporates mouse CD4 substitutions (Fig 8A). Phe^43^ is of interest because it is the primary contact for gp120. In previous studies an S42G/F43V CD4 D1D2 mutant was shown to fully abrogate CD4-gp120 interactions without disrupting the overall conformation of D1D2 [8,57]. We constructed an additional CD4 D2 mutant, K171D, that incorporates a substitution within the KIDIV sequence in the D2 G β-strand, expecting that it would disrupt ⍺_4_β_7_ binding and serve as a control. Wild type and mutant dodecamers were reacted with ⍺_4_β_7_ -expressing CD8^+^ T cells. D1D2-Ig⍺tp bound to ⍺_4_β_7_ in a dose dependent manner (Fig 8B). K171D D1D2-Ig⍺tp showed reduced binding to ⍺_4_β_7_. Of note, S42G/F43V-D1D2 -Igαtp showed an even greater reduction in reactivity, suggesting that a region of CD4 D1 that includes the C’C” strands and a connecting loop mediates the CD4-⍺_4_β_7_ interaction.

We next asked whether the CD4 D1 mAb Leu3A could block ⍺_4_β_7_ binding to CD4. Phe^43^ is a key residue in the Leu3A epitope, and Leu3A is a potent neutralizing antibody that blocks gp120 binding to CD4 [57]. To this end, we employed an SPR -based kinetic binding assay in which soluble ⍺_4_β_7_ was passed over surface immobilized CD4 D1D2, in the presence vs absence of Leu3A. Leu3A reduced ⍺_4_β_7_ binding to the same degree as preloading (blocking) the surface with soluble ⍺_4_β_7_ (Fig 8C). Ibalizumab, a CD4 D2 mAb, which we employed as a reagent control, failed to inhibit ⍺_4_β_7_ binding. Taken together these results indicate that, in addition to the G β-strand of D2, a region of D1 including Phe^43^ comprises a 2^nd^ region of CD4 that mediates ⍺_4_β_7_ binding.

Finally, we asked whether gp120, CD4 and ⍺_4_β_7_ can form a ternary complex. Labelled D1D2-Igαtp was bound to cell surface expressed ⍺_4_β_7_ in the presence vs absence of unlabeled gp120. With a 5X molar excess of gp120, we observed a >70% reduction in binding (Fig 8D). This finding is consistent with results presented in Fig 8B and suggests that binding sites on CD4, ⍺_4_β_7_ and gp120 utilize shared epitopes in a way that reduces the likelihood that all three proteins interact simultaneously.

We conclude that CD4, like MAdCAM-1 and VCAM-1 utilizes two sites to mediate ⍺_4_β_7_ interactions. Relative to MAdCAM-1 and VCAM-1, the two sites in CD4 are inverted, such that the 1° CD4 binding site, as defined by the I/LD cation binding motif, lies in D2, while the accessory site lies in D1 (Fig 4B). This inversion may reflect a cis interaction in the case of CD4 vs. trans interactions for MAdCAM-1 and VCAM-1. The way that HIV has incorporated elements of this interaction into its envelope, summarized in S2B Fig, raises several questions, that are discussed below.

## Discussion

In this study we identified a specific interaction between CD4, the HIV entry receptor, and ⍺_4_β_7_ an integrin that mediates lymphocyte trafficking to GALT. We found that the way these two receptors interact with each other is reflected in their individual interactions with HIV gp120. This finding supports the proposition that memory CD4^+^ T cells that co-express α_4_β_7_ play a specific and important role in HIV pathogenesis [58,59].

We previously reported that, on CD4^+^ T cells, CD4 and ⍺_4_β_7_ can appear sufficiently close to each other to interact [36]. Here we report the results of two assays, one based on SPR measurements of ⍺_4_β_7_ binding to CD4 D1D2, and a second based on binding of oligomerized sCD4 to cell surface ⍺_4_β_7_, that provide clear evidence of a specific interaction between these two receptors. We do not exclude the possibility that CD4 interacts with ⍺_4_β_7_ in trans, however the combination of proximity and affinity argues for a cis interaction. It is well established that cis interactions between integrins and leukocyte receptors can regulate immune responses [37]. Several of these interactions involve receptors that, like CD4, present amino-terminal Ig -like domains [40,42]. The potential for α_4_β_7_ to regulate CD4, or vice versa, is a subject worthy of future investigation, and may be relevant to efficacy of several α_4_ integrin antagonists widely used in the treatment of IBDs and MS.

The selective pressures that underlie HIV evolving the capacity to bind to and signal through two receptors that interact with each other remain to be determined. Because ⍺_4_β_7_ adopts an active conformation only under certain conditions it is possible that ⍺_4_β_7_ provides a means for HIV to target cells that are susceptible to productive infection. This possibility is consistent with studies reporting the preferential infection of ⍺_4_β_7_^high^ CD4^+^ T cells in vitro [36] and in vivo [33,60]. We know that ⍺_4_β_7_ is associated with migration to gut tissues, and thus gp120 affinity for ⍺_4_β_7_ could help explain the gut -tropic nature of HIV. Because CD4 migrates into the TCR in a transient way, where it plays an essential role in antigen recognition and the stabilization of immune synapses, we can speculate that interactions between CD4 and ⍺_4_β_7_ might regulate TCR activity. Perhaps HIV is exploiting this function to its own advantage. Finally, the close-proximity of ⍺_4_β_7_ to CD4 may impact viral entry and fusion. ⍺_4_β_7_ is not required for viral entry and cells lacking ⍺_4_β_7_ are clearly infected. However, productive infection is favored in activated cells, and gp120 delivers signals through ⍺_4_β_7_ [26,39]. Thus, engagement and signaling through ⍺_4_β_7_ either before or following CD4 binding may enhance infection in cells that are suboptimally activated. In this regard, integrins including ⍺_4_β_7_ mediate rearrangement of the cytoskeleton in the context of cellular activation [61].

Both MAdCAM-Ig and VCAM-Ig inhibit CD4 binding to ⍺_4_β_7_, indicating that CD4 engages the ligand binding groove in the ⍺_4_β_7_ headpiece. CD4-⍺_4_β_7_ interactions could only be detected in the presence of Mn^++^. Mn^++^ drives the extension of the ⍺_4_β_7_ leg domains in a way that places the headpiece >20nm above the cell surface. The N-terminus of CD4 appears no more than 12 nm above the cell surface. How then could CD4 engage the ⍺_4_β_7_ headpiece in cis? We recognize two possibilities. Upon cellular activation, both the TCR and CD4 selectively migrate toward the raised tips of microvilli [62]. As such, the contours of a T cell could provide a way for an extended form of ⍺_4_β_7_ to engage CD4 when CD4 appears above the plane of the cell membrane. If in fact CD4-⍺_4_β_7_ complexes are associated with specialized membrane structures, this could explain the selective pressures that resulted in a gp120 that mimics CD4-⍺_4_β_7_ interactions. Alternatively, some integrins headpieces can adopt an active conformation while their leg domains remain bent [49]. Current evidence indicates that the activated form of ⍺_4_β_7_ is extended [63], but perhaps the possibility of an additional form of ⍺_4_β_7_ that is activated but bent requires further investigation.

The structural similarities between CD4 D1D2, and the D1D2s of MAdCAM-1 and VCAM-1 suggest that CD4 might similarly utilize two sites (1° and accessory site) distributed across its two N-terminal domains to engage ⍺_4_β_7_. Peptides spanning residues 165–178 in D2, and 33–47 in D1 partially inhibited ⍺_4_β_7_-CD4 interactions. Mutating residues in either of these two regions abrogated ⍺_4_β_7_ binding in the same way that substitutions in either the 1° or accessory sites of MAdCAM-1 and VCAM-1 also eliminate ⍺_4_β_7_ reactivity. Their 1° binding sites reside in their respective D1 domains, and each includes an essential Asp-aliphatic aa motif (MAdCAM-1: LD, VCAM-1: ID) that coordinates a divalent cation. An analogous motif (I^172^D^173^) appears in CD4 D2, and binding of a D2 peptide that includes these two residues is Mn^++^ -dependent. We therefore assigned CD4 residues 165–178 as the 1° ⍺_4_β_7_ binding site.

The sequence of the CD4 1° binding site is related to gp120 V2 aa 170–184, which mediates cation -dependent binding of gp120 V2 to ⍺_4_β_7_. 8 out of 14 CD4 1° binding site residues align with conserved residues in the V2 mid-region. Notably, the CD4 sequence KIDIV, is nearly identical to the conserved V2 sequence KLDIV. We show that a gp120 V2 peptide (pV2^170-184^) inhibits ⍺_4_β_7_-CD4 interactions, while a CD4 D2 peptide (pCD4^165-178^) inhibits gp120 V2-⍺_4_β_7_ interactions. This reciprocal pattern of inhibition is consistent with molecular mimicry.

MAdCAM-1 and VCAM-1 encode distinct accessory α_4_β_7_ binding sites. Our findings indicate that the CD4 D1 C’C” strands and a connecting loop, also functions as an accessory binding site. Substituting residues Ser^42^ and Phe^43^ with corresponding mouse residues eliminated ⍺_4_β_7_ reactivity. These substitutions do not disrupt the overall antigenic structure of D1 [57], and a high-resolution crystal structure of D1D2 with an F^43^/V substitution found that this protein and w.t D1D2 were essentially isomorphous [64]. We conclude that the loss of ⍺_4_β_7_ reactivity mediated by these aa substitutions reflects a specific effect. Supporting this finding, mAb Leu3A, whose epitope includes Phe^43^ [57], inhibits D1D2 binding to ⍺_4_β_7_. Whether CD4 D1 mediates CD4-⍺_4_β_7_ interactions directly or indirectly is a subject of current investigation.

The sequence similarity between the α_4_β_7_ binding sites in CD4 D2 and gp120 V2 is relevant to HIV vaccine development insofar as this region of V2 played a central role in RV144, the only HIV vaccine trial to achieve efficacy. Reduced risk in RV144 correlated with weakly-neutralizing antibodies specific to a region of V2 encompassed by aa 169–181 [65–67]. Moreover, V2 residues, K^169^ and I^181^ were subject to vaccine -mediated immune pressure (sieving) in vaccinees who became infected [68]. I^181^ is included in the KIDIV sequence and K^169^ is adjacent to Q^170^K^171^. These observations indicate that the immune responses associated with reduced risk in RV144 target a region of gp120 V2 that mimics the 1° ⍺_4_β_7_ binding site in CD4 D2. Additionally, the primary contact sites of two RV144 -derived mAbs, CH58 and CH59, that are considered representative of the relevant RV144 Ab response lie within V2 aa 170–184 [69–71]. These mAbs block V2 adhesion to ⍺_4_β_7_ [29,56,71]. The prevailing view holds that reduced risk in RV144 was associated with V2 Ab effector functions [72]. We wonder whether the mechanistic explanations of RV144 reduced risk require further refinement. Such information may contribute to our understanding of the failure of HVTN702, a vaccine regimen intended to elicit V1V2 antibodies [73]. Overall, our findings provide an additional rationale for continuing to investigate the potential utility of V2 antibodies as part of an efficacious HIV vaccine.

The limitations of this study include a recognition that we do not know how the direct interaction between α_4_β_7_ and CD4 impacts HIV infection. However, the presence of a sequence in V2 that mimics CD4 D2 and shares with it the capacity to directly bind α_4_β_7_ provides strong evidence that HIV is sensitive, in some way, to α_4_β_7_-CD4 interactions. This conclusion is consistent with the preferential infection in vivo of memory CD4^+^ T cells that co-express CD4 and ⍺_4_β_7_ [33]. Depending on whether CD4-⍺_4_β_7_ interactions enhance or inhibit infection, it follows that circumstances that promote this interaction define a condition in which CD4^+^ T cells are either more or less susceptible to productive infection. Because productive infection is strongly impacted by the activation state of a cell, determining if and how CD4-⍺_4_β_7_ interactions regulate CD4 participation in TCR-MHC II interactions may help resolve this question. Additionally, although not the subject of this study, our understanding of how gp120 interacts with ⍺_4_β_7_ is also incomplete, but relevant to the findings presented here. In contrast to the 1° ⍺_4_β_7_ binding site in CD4 D2, which is well exposed [10], the analogous ⍺_4_β_7_ binding site in gp120 V2 is partially hidden in the predominant ground state represented in prefusion trimers [13,74–78]. We do not know at which step in the transition of gp120 from the closed trimer to the open coreceptor bound state that V2 interacts with ⍺_4_β_7_, nor whether this interaction precedes or follows CD4 interactions with gp120. Perhaps a more detailed understanding of V2 conformation as the trimer moves away from its ground state will resolve the context in which gp120 engages ⍺_4_β_7_ [14,78].

In conclusion, in this report we identify two new aspects of the CD4 receptor and the way that it interacts with gp120 that are relevant to HIV pathogenesis. First, CD4 binds directly to ⍺_4_β_7_, the integrin most closely associated with T cell trafficking to GALT. Second, gp120 interactions with CD4 and ⍺_4_β_7_ reflect their interactions with each other. These findings underscore the view that ⍺_4_β_7_ -expressing CD4^+^ T cells play a specific and important role in HIV pathogenesis.

## Materials and methods

### Ethics statement

All primary T cells utilized in these studies were isolated from PBMCs, collected from healthy donors through a NIH Department of Transfusion Medicine protocol that was approved by the Institutional Review Board of the National Institute of Allergy and Infectious Diseases (NIAID), National Institutes of Health. Informed consent was written and was provided to study participants.

### Cell line and reagents

RPMI8866 cells, a human B lymphoma cell line that constitutively expresses α_4_β_7_ was purchased from Sigma-Aldrich. Cells were maintained in RPMI-1640 (Gibco) containing 10% heat inactivated fetal bovine serum (Gibco), 2% penicillin/streptomycin/glutamine (Gemini Bio-Products) and 0.1% all trans RA (Sigma). Cells were cultured for a minimum of 7 days prior to use. Primary CD4^+^ and CD8^+^ T cells were purified from buffy coats by negative bead selection. Cells were cultured in RPMI-1640 plus anti CD3, IL-2, and RA for a minimum of 8 days prior to use. CD4 D1D2 protein was a gift from Dr. E. Berger. Linear CD4 peptides and FAM conjugated peptides with > 90% purity were provided by Biopeptide. Cyclic V2 peptides were supplied by JPT Peptide Technologies. α_4_ and β_7_ expression vectors were purchased from Origene Technologies. Synthetic CD4 D1D2 and D1D2-Ig⍺tp genes were purchased from ATUM. Site directed mutagenesis was performed by Genscript. Transient transfections were carried out with Polyfect (Invitrogen), using standard protocols. CHO cell derived A244 gp120 (Lot 26539–1) was provided by Global Solutions for Infectious Diseases (San Francisco, CA). Purity was estimated at 97.1%. Vedolizumab was provided by the NIH Clinical Center Pharmacy Department. FG was purchased from MedChem Express. Human integrin α_4_ mAb 2B4, MAdCAM-Ig, VCAM-Ig and soluble α_4_β_7_ were purchased from R&D Systems. Ibalizumab was generously provide by Dr. David Ho. Other mAbs were purchased from BD Biosciences. Dye labelling of D1D2-Ig⍺tp was carried out using a LYNX HRP conjugation kit (Bio-Rad), following the manufacturer’s instructions.

### Flow cytometry binding assays

Cells were stained with fluoresceine conjugated mAbs or recombinant proteins using standard procedures preceded by Fc receptor blocking with human IgG. Buffers used were 10 mM HEPES 150 mM NaCl (HBS Buffer) with 100 μM CaCl_2_. 1 mM MnCl_2_ was also included, unless otherwise specified. Buffer without divalent cations included 0.1 mM EDTA. Unlabeled mAb or protein inhibitors were preincubated with cells or proteins at a 5X molar excess for 15 min on ice. FAM labeled peptides were used at 1.25 μg/ 200,000 cells in a 100 μl volume. Data were acquired using a FACSCanto II (BD Biosciences) and analyzed with FlowJo software (BD Biosciences). Plotted MFI values represent absolute values.

### Surface plasmon resonance

Experiments were performed using a Biacore 3000 instrument (Cytiva Life Sciences) using CM4 or CM5 sensor chips. The data were evaluated using BIAevaluation 4.1.1 software (Cytiva Life Sciences). The chip surface was activated by injecting 35 μl of a 1:1 mixture of 0.05 M *N*-hydroxysuccinimide and 0.2 M *N*-ethyl-*N*-(dimethylaminopropyl) carbodiimide at 5 μl/min. NeutrAvidin, HIV gp120, sCD4 (D1D2) or Hu-MAdCAM-Ig (R&D Systems) at concentrations of 5 μg/ml in 10mM NaOAc, pH 4.5, were immobilized to 250–750 resonance units (RU). After the proteins were immobilized to the desired densities, unreacted sites were blocked with 35 μl of 1 M Tris-HCl (pH 8.0). Biotinylated cyclic V2 peptides (1 μg/ml in 20 mM Tris-HCl, pH 8.0) were bound to the NeutrAvidin surfaces to densities of approximately 250–300 RU. One surface was activated and blocked without ligand as a control surface for non-specific binding. Signal from the control surface was subsequently subtracted. Running buffer was HBS (pH 7.4), 1 mM MnCl_2_, 0.005% Tween- 20, 0.05% soluble carboxymethyl-dextran. Binding experiments were carried out at a flow rate of 25 μl/min at 25°C. After a 2 min injection, the surface was washed for an additional 2 min in running buffer allowing dissociation of the bound ligand from the surface. The surfaces were regenerated by multiple injections of 4.5 M MgCl_2_ at a flow rate of 100 μl/min. Inhibition of ⍺_4_β_7_ binding by linear V2-loop and CD4 derived peptides was carried out by pre-incubating ⍺_4_β_7_ with the peptides in running buffer at the indicated concentrations for 2 hours at room temperature prior to passing them over the prepared surfaces as described above. Cross-inhibition experiments were performed by passing either buffer or the listed analytes over a surface to which sCD4 (D1D2) had been immobilized, followed by a secondary sequential injection of ⍺_4_β_7_. The ability of each analyte to inhibit either itself or ⍺_4_β_7_ was determined by comparing the binding of ⍺_4_β_7_ in the absence or presence of the listed pre-bound analytes as previously described [79]. Illustration of the dynamic range of range of linear peptide inhibition assays is provided in S4 Fig.

### FRET/FLIM

Primary CD4^+^ T cells were cultured in IL-2 + anti CD3 + RA (10nM) for a minimum of 10 days. RA -mediated upregulation of ⍺_4_β_7_ was verified by flow cytometry prior to mAb staining and FRET\FLIM Imaging. FLIM sample staining was carried out 2 days after fresh media exchange. Samples were reacted with the following antibodies using standard immunofluorescence methods. Donor mAbs (AF488 conjugated) includes OKT4 (BioLegend 317420), FIB504 (lab conjugated), Leu3A (Biolegend 344618). Acceptor mAbs (AF546 conjugated) includes FIB504 (lab conjugated), OKT4 (lab conjugated). Mock and gp120 treatments were performed in the same volume of PBS (cells: 2x10^6^/mL), for 20 or 60 min at 37°C in the dark, in the presence of 5% CO_2_. Treatments were stopped by 4 vigorous washes in high volumes (3x) of ice-cold complete media, followed by 1 wash in PBS + BSA, then fixed in 2% MeOH-free PFA at 4C. After the completion of the staining procedure (~16 hrs) samples were cytospun onto imaging slides at 400 rpm for 6 min. Slides were then mounted with imaging coverslips using prolong gold mounting media with antifade-no DAPI (Invitrogen) and cured thoroughly in the dark at room temperature before imaging. Images were acquired using a Leica DMI6000 confocal microscope coupled with Becker and Hickel Lifetime measurement module. Data were collected in excess of 1000 photons per pixel to fit the curve in a multi-modal decay plot. Images were post-processed using ImageJ/NIHimage analysis software.

### Proximity ligation assay

Flow cytometry based PLA assays (Sigma Aldrich) were carried out per the manufacturer’s instructions. In brief, an oligonucleotide conjugated anti CD4 mAb (clone DB81) (plus strand) and an oligonucleotide conjugated anti integrin β_7_ mAb (clone FIB27) (minus strand) were generated using a Duolink In Situ Probemaker kit. Freshly isolated purified CD4^+^ T cells were fixed in 1% PFA and blocked with buffer containing 2% donkey serum, 10% BSA and 0.2% gelatin. Cells were incubated with mAb probes for 30 min at 37°C in buffer containing 0.2% donkey serum, 10% BSA and 0.2% gelatin. Additional fluorescein based mAbs (vedolizumab and CD45RA) were included. After washing, cells were treated with DNA ligase and Duolink ligation buffer for 30 min at 37°C. After washing, cells were treated with Duolink amplification buffer and DNA polymerase O/N at 37°C. Cells were washed and treated with Duolink detection buffer (Far Red) for 30 min at 37°C. After washing cells were resuspended in PBS and analyzed by flow-cytometry. All washes were carried in Duolink wash buffer.

### CD4 and gp120 V2 sequence alignments and analysis

All gp120 aa sequences were numbered based on HXB2. Frequencies of gp120 V2 aa were established by accessing the HIV ENV web alignment from Los Alamos database (https://www.hiv.lanl.gov/content/sequence/NEWALIGN/align.html (accession date 09/29/2022)). Sequences from different HIV subtypes and recombinants were utilized and represent the full spectrum of HIV envelope sequences from the LANL database. All non-group M sequences were excluded from this analysis. Geneious 11.1.5 software was used to determine the frequencies of I, L and V at gp120 position 181. HIV ENV web alignment was used to generate the logo figure on WebLogo3 software (DOI: 10.1101/gr.849004). Protein motifs were scanned against the Swiss-Prot database using the Prosite tool (doi:10.1093/nar/gks1067, https://doi.org/10.1093/nar/gkl124).

## Data and materials availability

All data are available in the main text or the supplementary materials.

## Supporting information

S1 Fig Dynamic association of CD4 and ⍺_4_β_7_ on ⍺_4_β_7_^high^ CD4^+^ T cells. (A) Schematic of trans vs cis interactions of integrins with counter receptors. (B) PLA assay for CD4 and Integrin β_7_ with PLA antibody probes (CD4 mAb (plus strand) and β_7_ mAb (minus strand). Representative flow cytometric dot plot of freshly isolated primary CD4^+^ T cells stained with vedolizumab (Y-axis) and CD45RA (X-axis). ⍺_4_β_7_^high^ (yellow), ⍺_4_β_7_^int^ (blue), and ⍺_4_β_7_^low^ (green) gates are shown, with cell population frequencies depicted (middle). Pie charts (right) indicate the frequency of PLA positive cells (red) within the ⍺_4_β_7_^high^, ⍺_4_β_7_^int^, and ⍺_4_β_7_^low^ gates. (C) FLIM analysis of ⍺_4_β_7_^high^ CD4^+^ T cells stained with dye labeled anti CD4 (donor) and anti β_7_ (acceptor) mAbs. Cells mock treated and stained with CD4 mAb alone (green). Cells stained with CD4 and β_7_ mAbs following mock treatment (black), gp120 treated for 20 min (blue), or gp120 treated for 60 min (red). Number of cells analyzed as indicated. Y-axis and X-axis indicate normalized frequency and lifetime (picoseconds) respectively. (D) Representative images of cells stained with CD4 alone (left), CD4 and β_7_ mAbs following mock treatment (center), or CD4 and β_7_ mAbs following 60 min gp120 treatment (right). Color bar represents heatmap for the interaction in picoseconds.
(TIF)

S2 Fig Potential regulatory role of a CD4-⍺_4_β_7_ cis interaction. (A) Cis interaction between CD4 D1D2 and the headpiece domain of α_4_β_7_. (B) Schematic summarizing the interactions of CD4 with gp120 and α_4_β_7_.
(TIF)

S3 Fig CD4 D2 aa 165–178 sequence conservation in nonhuman primates. Amino acids 165–178 in nonhuman primate CD4 orthologs. Residues that align with conserved gp120 V2 residues 170–184 are shaded in grey.
(TIF)

S4 Fig Linear peptide inhibition of D1D2-⍺_4_β_7_ interactions by SPR. Recombinant α_4_β_7_ (analyte) reacted with surface bound CD4 D1D2 in the absence or presence of increasing concentrations of peptide pCD4^165-175^. Y-axis indicates relative mass units (RU) (left). Peak RU at the termination of the association phase (150s), plotted against peptide concentration (right).
(TIF)

S5 Fig Inhibition of D1D2-⍺_4_β_7_ interactions by New- and Old-World monkey CD4 peptides. SPR assays of recombinant α_4_β_7_ (analyte) reacted with surface bound CD4 D1D2 in the absence or presence of increasing concentrations of Gorilla (left) and Aotus (right) analogs of peptide pCD4^165-175^. Y-axis indicates relative mass units (RU) (upper). Amino acid sequence alignment of Gorilla and Aotus pCD4^165-175^ analogs (lower). Dots indicate identity.
(TIF)

S1 Table ⍺_4_β_7_ binding kinetics. (DOCX)

S2 Table CD4 peptides amino acids +1(23)-188*. (DOCX)

S3 Table Proteins encoding CD4 1° binding site motifs. (DOCX)
