# Supplementary figures and images for: The V2 domain of HIV gp120 mimics an interaction between CD4 and integrin ⍺4β7
Source: PLoS Pathog. 2023 Dec 8;19(12):e1011860. doi: 10.1371/journal.ppat.1011860 (PMC10732398; doi:10.1371/journal.ppat.1011860)

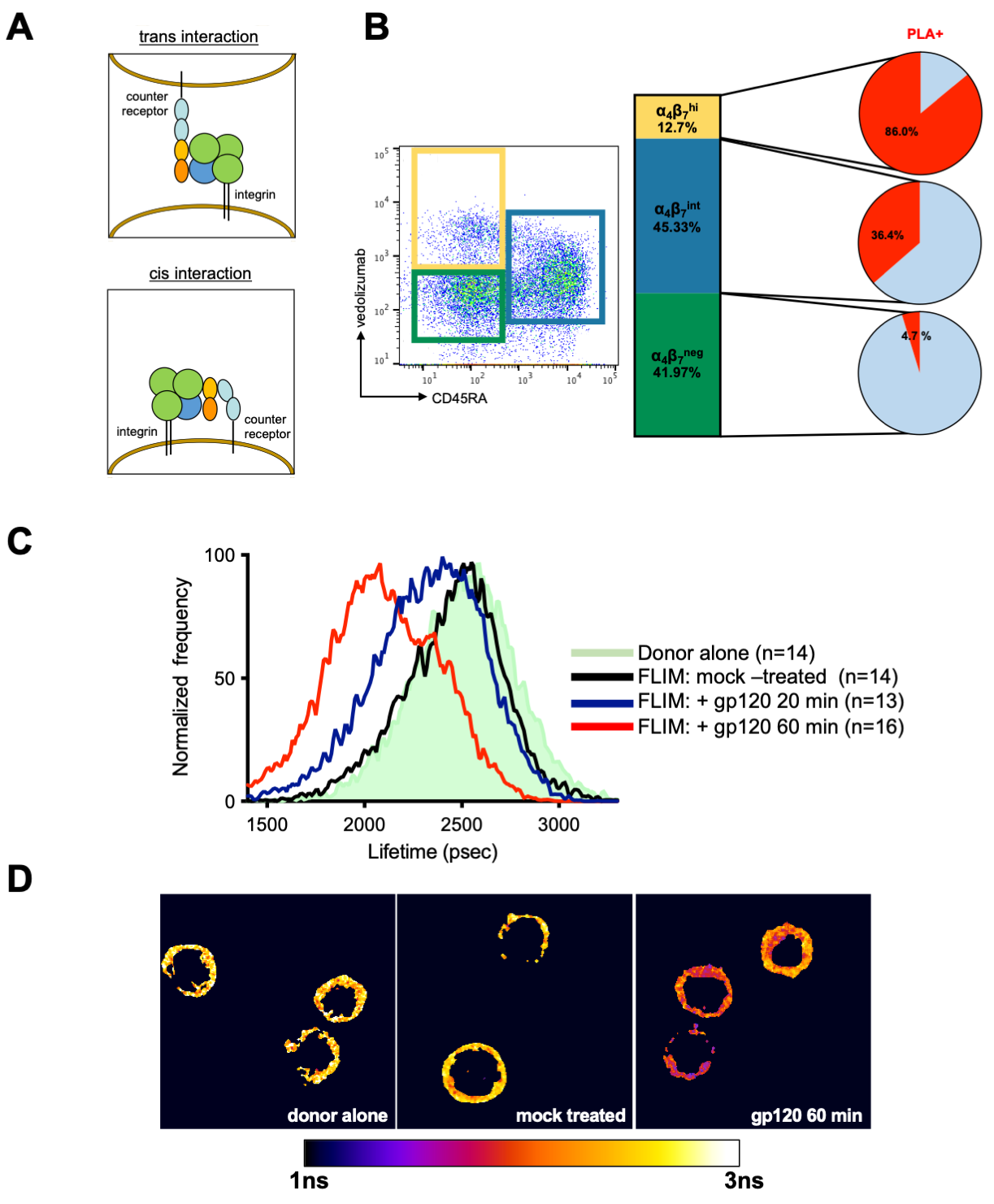

Supplement: S1 Fig — (A) Schematic of trans vs cis interactions of integrins with counter receptors. (B) PLA assay for CD4 and Integrin β7 with PLA antibody probes (CD4 mAb (plus strand) and β7 mAb (minus strand). Representative flow cytometric dot plot of freshly isolated primary CD4+ T cells stained with vedolizumab (Y-axis) and CD45RA (X-axis). ⍺4β7high (yellow), ⍺4β7int (blue), and ⍺4β7low (green) gates are shown, with cell population frequencies depicted (middle). Pie charts (right) indicate the frequency of PLA positive cells (red) within the ⍺4β7high, ⍺4β7int, and ⍺4β7low gates. (C) FLIM analysis of ⍺4β7high CD4+ T cells stained with dye labeled anti CD4 (donor) and anti β7 (acceptor) mAbs. Cells mock treated and stained with CD4 mAb alone (green). Cells stained with CD4 and β7 mAbs following mock treatment (black), gp120 treated for 20 min (blue), or gp120 treated for 60 min (red). Number of cells analyzed as indicated. Y-axis and X-axis indicate normalized frequency and lifetime (picoseconds) respectively. (D) Representative images of cells stained with CD4 alone (left), CD4 and β7 mAbs following mock treatment (center), or CD4 and β7 mAbs following 60 min gp120 treatment (right). Color bar represents heatmap for the interaction in picoseconds. (TIF) [file ppat.1011860.s001.tif]

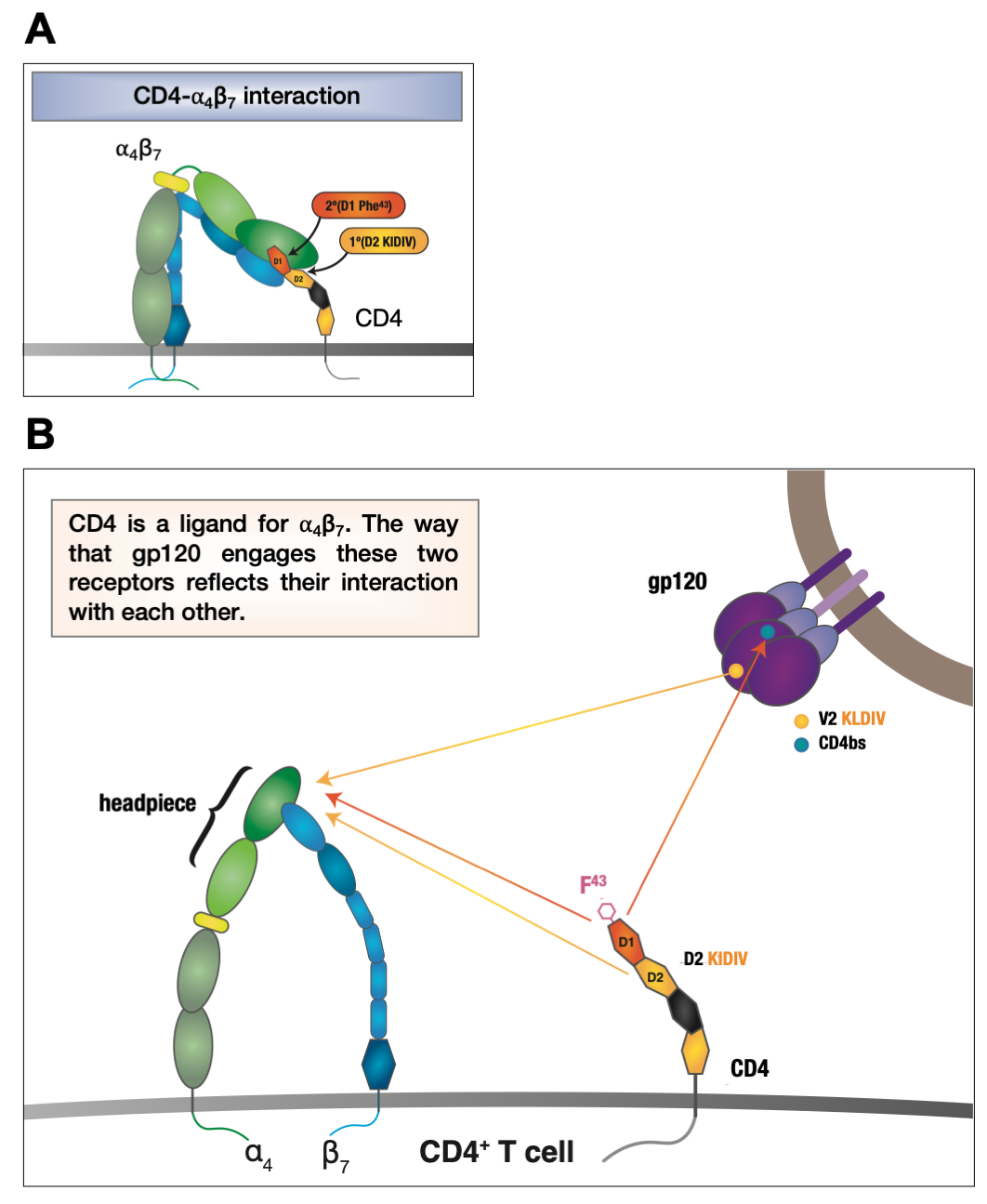

Supplement: S2 Fig — (A) Cis interaction between CD4 D1D2 and the headpiece domain of α4β7. (B) Schematic summarizing the interactions of CD4 with gp120 and α4β7. (TIF) [file ppat.1011860.s002.tif]

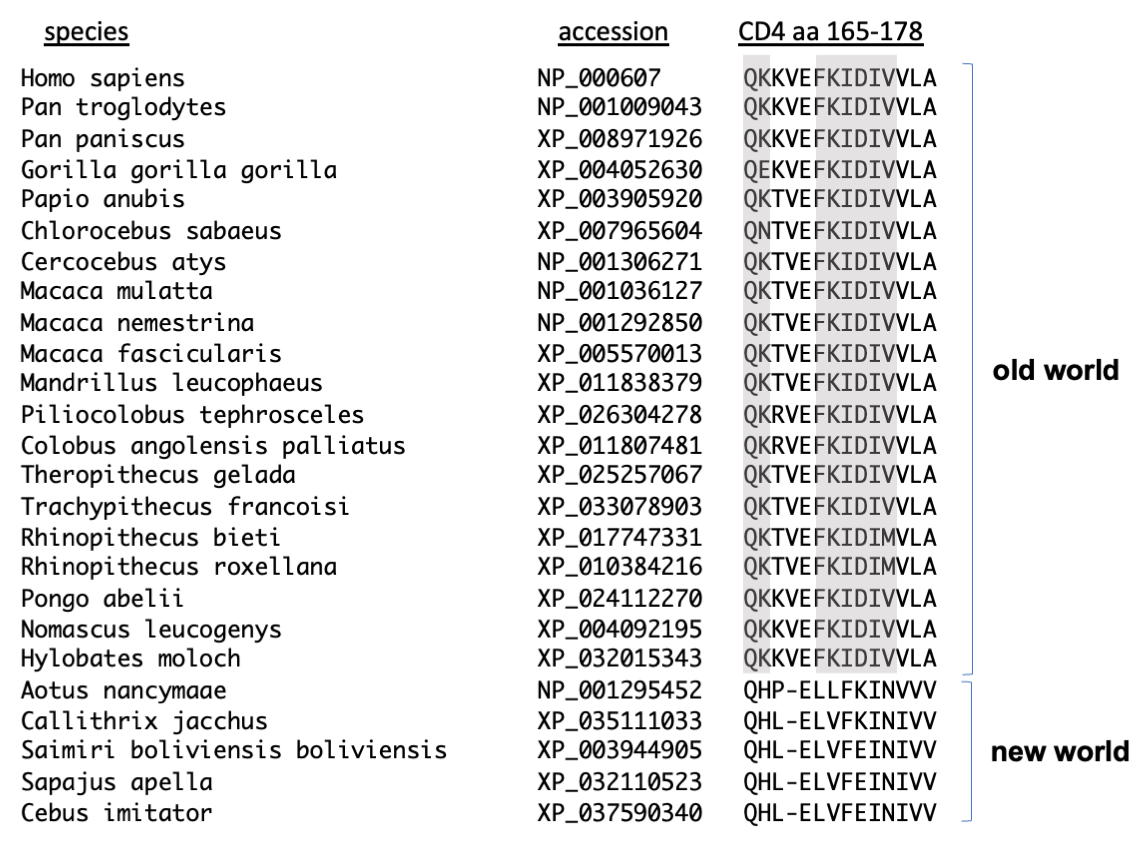

Supplement: S3 Fig — Amino acids 165–178 in nonhuman primate CD4 orthologs. Residues that align with conserved gp120 V2 residues 170–184 are shaded in grey. (TIF) [file ppat.1011860.s003.tif]

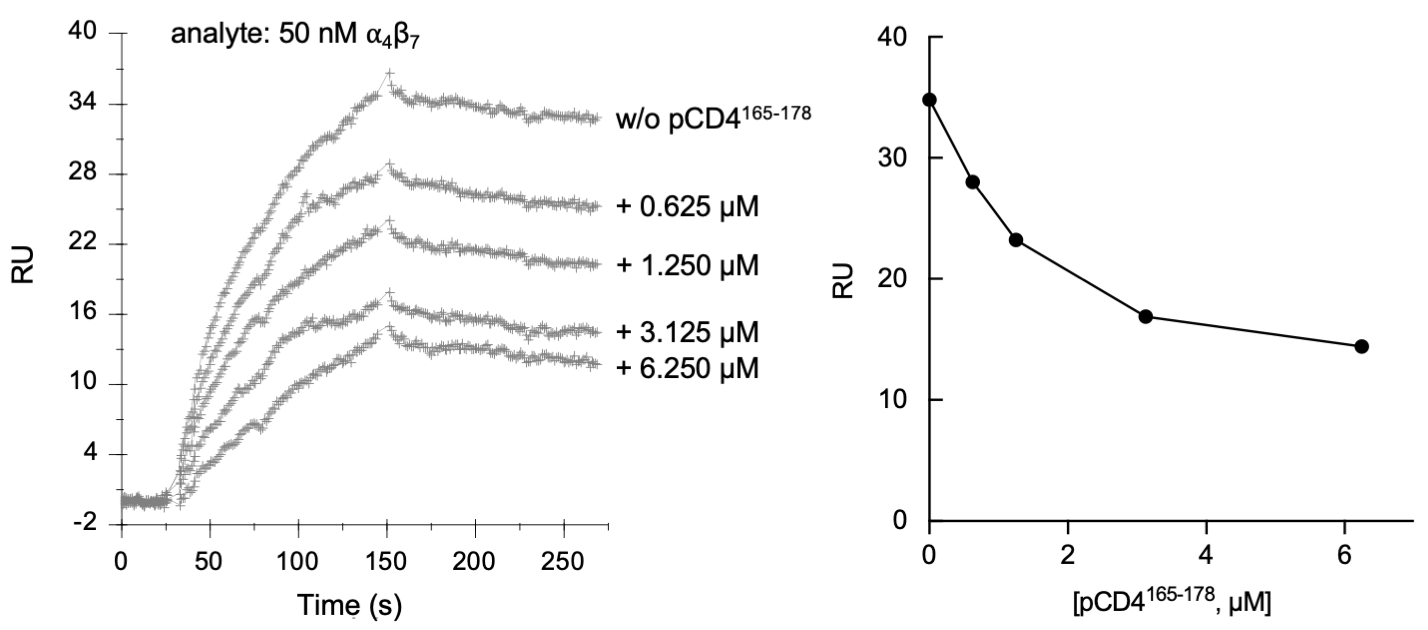

Supplement: S4 Fig — Recombinant α4β7 (analyte) reacted with surface bound CD4 D1D2 in the absence or presence of increasing concentrations of peptide pCD4165-175. Y-axis indicates relative mass units (RU) (left). Peak RU at the termination of the association phase (150s), plotted against peptide concentration (right). (TIF) [file ppat.1011860.s004.tif]

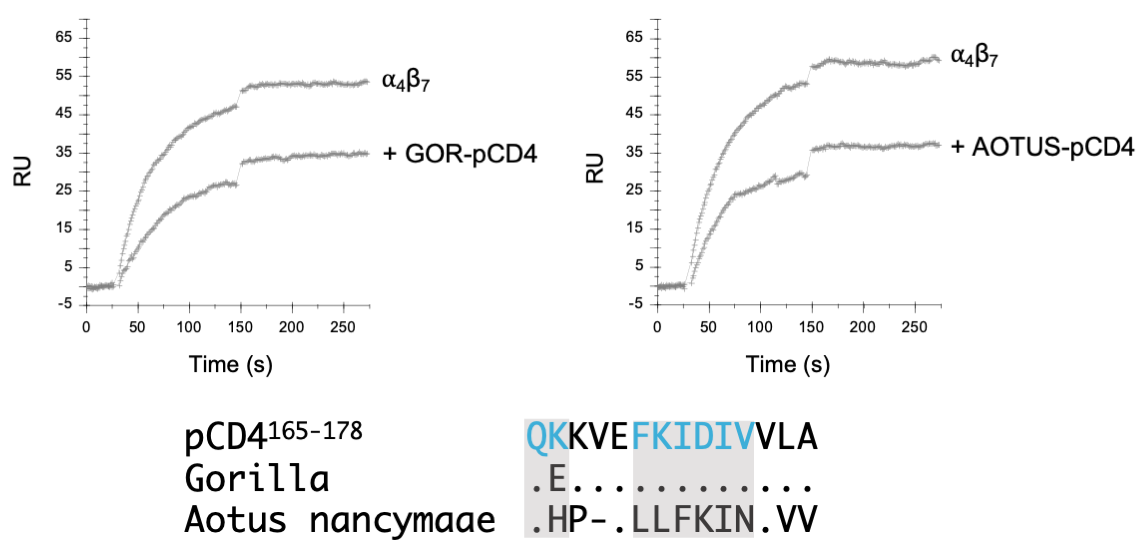

Supplement: S5 Fig — SPR assays of recombinant α4β7 (analyte) reacted with surface bound CD4 D1D2 in the absence or presence of increasing concentrations of Gorilla (left) and Aotus (right) analogs of peptide pCD4165-175. Y-axis indicates relative mass units (RU) (upper). Amino acid sequence alignment of Gorilla and Aotus pCD4165-175 analogs (lower). Dots indicate identity. (TIF) [file ppat.1011860.s005.tif]
