# Supplementary material for: The V2 domain of HIV gp120 mimics an interaction between CD4 and integrin ⍺4β7
Source: PLoS Pathog. 2023 Dec 8;19(12):e1011860. doi: 10.1371/journal.ppat.1011860 (PMC10732398; doi:10.1371/journal.ppat.1011860)
Supplement: S1 Table — (DOCX) [file ppat.1011860.s006.docx]

**S1 Table. ⍺_4_β_7_ binding kinetics**

|  | **MAdCAM-Ig** | **VCAM-Ig** | **D1D2** | **BG505**  **gp120** | **BG505**  **SOSIP** |
| --- | --- | --- | --- | --- | --- |
| ka (1/Ms) | 9.67E+04 | 3.87E+04 | 1.30E+05 | 1.37E+06 | 8.40E+05 |
| kd (1/s) | 8.63E-04 | 1.41E-03 | 4.64E-04 | 1.36E-03 | 1.12E-03 |
| KA (1/M) | 1.12E+08 | 2.76E+07 | 2.80E+08 | 1.01E+09 | 7.47E+08 |
| KD (M) | 8.92E-09 | 3.63E-08 | 3.57E-09 | 9.94E-10 | 1.34E-09 |
| Chi2 | 1.83E+00 | 5.01E-01 | 5.98E-01 | 1.29E+00 | 1.20E+00 |
