# Supplementary material for: The V2 domain of HIV gp120 mimics an interaction between CD4 and integrin ⍺4β7
Source: PLoS Pathog. 2023 Dec 8;19(12):e1011860. doi: 10.1371/journal.ppat.1011860 (PMC10732398; doi:10.1371/journal.ppat.1011860)
Supplement: S2 Table — (DOCX) [file ppat.1011860.s007.docx]

**S2 Table. CD4 peptides amino acids +1(23)-188***

| **CD4 aa** | ***** | **Sequences** |
| --- | --- | --- |
| CD4 P1 |  | KKVVLGKKGDTVELT |
| CD4 P2 | C12A | LGKKGDTVELTATAS |
| CD4 P3 | C8A | GDTVELTATASQKKS |
| CD4 P4 | C4A | ELTATASQKKSIQFH |
| CD4 P5 |  | TASQKKSIQFHWKNS |
| CD4 P6 |  | KKSIQFHWKNSNQIK |
| CD4 P7 |  | QFHWKNSNQIKILGN |
| CD4 P8 |  | KNSNQIKILGNQGSF |
| CD4 P9 |  | QIKILGNQGSFLTKG |
| CD4 P10 |  | LGNQGSFLTKGPSKL |
| CD4 P11 |  | GSFLTKGPSKLNDRA |
| CD4 P12 |  | TKGPSKLNDRADSRR |
| CD4 P13 |  | SKLNDRADSRRSLWD |
| CD4 P14 |  | DRADSRRSLWDQGNF |
| CD4 P15 |  | SRRSLWDQGNFPLII |
| CD4 P16 |  | LWDQGNFPLIIKNLK |
| CD4 P17 |  | GNFPLIIKNLKIEDS |
| CD4 P18 |  | LIIKNLKIEDSDTYI |
| CD4 P19 | C12A | NLKIEDSDTYIAEVE |
| CD4 P20 | C8A | EDSDTYIAEVEDQKE |
| CD4 P21 | C4A | TYIAEVEDQKEEVQL |
| CD4 P22 |  | EVEDQKEEVQLLVFG |
| CD4 P23 |  | QKEEVQLLVFGLTAN |
| CD4 P24 |  | VQLLVFGLTANSDTH |
| CD4 P25 |  | VFGLTANSDTHLLQG |
| CD4 P26 |  | TANSDTHLLQGQSLT |
| CD4 P27 |  | DTHLLQGQSLTLTLE |
| CD4 P28 |  | LQGQSLTLTLESPPG |
| CD4 P29 |  | SLTLTLESPPGSSPS |
| CD4 P30 | C14A | TLESPPGSSPSVQAR |
| CD4 P31 | C10A | PPGSSPSVQARSPRG |
| CD4 P32 | C6A | SPSVQARSPRGKNIQ |
| CD4 P33 | C2A | QARSPRGKNIQGGKT |
| CD4 P34 |  | PRGKNIQGGKTLSVS |
| CD4 P35 |  | NIQGGKTLSVSQLEL |
| CD4 P36 |  | GKTLSVSQLELQDSG |
| CD4 P37 | C15A | SVSQLELQDSGTWTA |
| CD4 P38 | C11A | LELQDSGTWTATVLQ |
| CD4 P39 | C7A | DSGTWTATVLQNQKK |
| CD4 P40 | C3A | WTATVLQNQKKVEFK |
| CD4 P41 |  | VLQNQKKVEFKIDIV |
| CD4 P42 |  | QKKVEFKIDIVVLA |
| CD4 P43 |  | VEFKIDIVVLAFQK |
| CD4 P44 |  | KIDIVVLAFQKASS |
| CD4 P45 |  | IVVLAFQKASSIVY |

*cysteines substituted with alanines
