# Supplementary material for: The V2 domain of HIV gp120 mimics an interaction between CD4 and integrin ⍺4β7
Source: PLoS Pathog. 2023 Dec 8;19(12):e1011860. doi: 10.1371/journal.ppat.1011860 (PMC10732398; doi:10.1371/journal.ppat.1011860)
Supplement: S3 Table — (DOCX) [file ppat.1011860.s008.docx]

**S3 Table. Proteins encoding CD4 1° binding site motifs**

| **Observed hits (Q-K-x(3)-F-x-[LI]-D-[IV]-V)** | | |
| --- | --- | --- |
| **Protein** | **Species** | **Motif** |
| T-cell surface glycoprotein CD4 | *Cercocebus atys* | QKtveFkIDI |
| T-cell surface glycoprotein CD4 | *Homo sapiens* | QKkveFkIDI |
| T-cell surface glycoprotein CD4 | *Macaca fascicularis* | QKtveFkIDI |
| T-cell surface glycoprotein CD4 | *Macaca fuscata fuscata* | QKtveFkIDI |
| T-cell surface glycoprotein CD4 | *Macaca mulatta* | QKtveFkIDI |
| T-cell surface glycoprotein CD4 | *Macaca nemestrina* | QKtveFkIDI |
| T-cell surface glycoprotein CD4 | *Pan troglodytes* | QKkveFkIDI |

| **Observed hits (K-I-D-I-V)** | | |
| --- | --- | --- |
| **Protein** | **Species** | **Motif** |
| GDP-Man:Man(3)GlcNAc(2)-PP-Dol alpha-1,2-mannosyltransferase | *Saccharomyces cerevisiae* | K-I-D-I-V |
| Protein BIG1 | *Kluyveromyces lactis* | K-I-D-I-V |
| T-cell surface glycoprotein CD4 | *Cercocebus atys* | K-I-D-I-V |
| T-cell surface glycoprotein CD4 | *Erythrocebus patas* | K-I-D-I-V |
| T-cell surface glycoprotein CD4 | *Homo sapiens* | K-I-D-I-V |
| T-cell surface glycoprotein CD4 | *Macaca fascicularis* | K-I-D-I-V |
| T-cell surface glycoprotein CD4 | *Macaca fuscata fuscata* | K-I-D-I-V |
| T-cell surface glycoprotein CD4 | *Macaca mulatta* | K-I-D-I-V |
| T-cell surface glycoprotein CD4 | *Macaca nemestrina* | K-I-D-I-V |
| T-cell surface glycoprotein CD4 | *Pan troglodytes* | K-I-D-I-V |
| Cadherin-rel. family member 1 | *Bos taurus* | K-I-D-I-V |
| Protein CWH43 | *Saccharomyces cerevisiae* | K-I-D-I-V |
| Diacylglycerol O-acyltransferase 1 | *Dictyostelium discoideum* | K-I-D-I-V |
| Deoxyhypusine synthase | *Yarrowia lipolytica* | K-I-D-I-V |
| Eukaryotic elongation factor 2 kinase | *Caenorhabditis elegans* | K-I-D-I-V |
| Fatty acid synthase subunit beta | *Candida albicans* | K-I-D-I-V |
| Guanine nucleotide-binding protein G(s) subunit alpha | *Xenopus laevis* | K-I-D-I-V |
| 5-aminolevulinate synthase, erythroid-specific, mitochondrial | *Brachydanio rerio* | K-I-D-I-V |
| 5-aminolevulinate synthase, erythroid-specific, mitochondrial | *Opsanus tau* | K-I-D-I-V |
| Heat shock cognate protein HSP 90-beta | *Gallus gallus* | K-I-D-I-V |
| Interleukin-1 receptor type 1 | *Homo sapiens* | K-I-D-I-V |
| Interleukin-1 receptor-like 2 | *Homo sapiens* | K-I-D-I-V |
| ATP-dependent DNA helicase 2 subunit KU80 | *Arabidopsis thaliana* | K-I-D-I-V |
| Probable endonuclease LCL3 | *Kluyveromyces delphensis* | K-I-D-I-V |
| Polycystin-1 | *Caenorhabditis elegans* | K-I-D-I-V |
| Probable polyketide synthase 2 | *Dictyostelium discoideum* | K-I-D-I-V |
| Putrescine N-methyltransferase 1 | *Nicotiana attenuata* | K-I-D-I-V |
| Putrescine N-methyltransferase 1 | *Nicotiana tabacum* | K-I-D-I-V |
| Putrescine N-methyltransferase 2 | *Nicotiana attenuata* | K-I-D-I-V |
| Putrescine N-methyltransferase 2 | *Nicotiana tabacum* | K-I-D-I-V |
| Putrescine N-methyltransferase 3 | *Nicotiana attenuata* | K-I-D-I-V |
| Putrescine N-methyltransferase 3 | *Nicotiana tabacum* | K-I-D-I-V |
| Putrescine N-methyltransferase 4 | *Nicotiana tabacum* | K-I-D-I-V |
| Bifunctional purine biosynthesis protein ade10 | *Schizosaccharomyces pombe* | K-I-D-I-V |
| Polyol phosphate phosphatase PYP1 | *Saccharomyces cerevisiae* | K-I-D-I-V |
| Ras-related protein RABA4a | *Arabidopsis thaliana* | K-I-D-I-V |
| Short-chain dehydrogenase/reductase SDRA | *Arabidopsis thaliana* | K-I-D-I-V |
| dTDP-D-glucose 4,6-dehydratase | *Bos taurus* | K-I-D-I-V |
| dTDP-D-glucose 4,6-dehydratase | *Dictyostelium discoideum* | K-I-D-I-V |
| dTDP-D-glucose 4,6-dehydratase | *Homo sapiens* | K-I-D-I-V |
| dTDP-D-glucose 4,6-dehydratase | *Mus musculus* | K-I-D-I-V |
| DNA topoisomerase 2 | *Trypanosoma cruzi* | K-I-D-I-V |
| Probable E3 ubiquitin-protein ligase DDB_G0283893 | *Dictyostelium discoideum* | K-I-D-I-V |
| FHA domain-containing protein At4g14490 | Arabidopsis thaliana | K-I-D-I-V |
